# Efficacy and safety of prone position in COVID-19 patients with respiratory failure: a systematic review and meta-analysis

**DOI:** 10.1186/s40001-022-00953-z

**Published:** 2022-12-27

**Authors:** Hyeon-Jeong Lee, Junghyun Kim, Miyoung Choi, Won-Il Choi, Joonsung Joh, Jungeun Park, Joohae Kim

**Affiliations:** 1 Division of Healthcare Technology Assessment Research, National Evidence-Based Healthcare Collaborating Agency, Seoul, South Korea; 2 Division of Pulmonary and Critical Care Medicine, Department of Internal Medicine, National Medical Center, Seoul, South Korea; 3 Division of Pulmonary and Allergy, Department of Internal Medicine, Hallym University Dongtan Sacred Heart Hospital, Hallym University College of Medicine, Hwaseong, Gyeonggi-do South Korea; 4 Department of Internal Medicine, Myongji Hospital, Hanyang University College of Medicine, Goyang, Gyeonggi-do South Korea

**Keywords:** Prone position, COVID-19, Acute respiratory distress syndrome

## Abstract

**Background:**

Prone position has already been demonstrated to improve survival in non-COVID acute respiratory distress syndrome and has been widely performed in COVID-19 patients with respiratory failure, both in non-intubated and intubated patients. However, the beneficial effect of the prone position in COVID-19 pneumonia still remains controversial. Therefore, we aimed to evaluate the effectiveness and safety of the prone position compared with the non-prone in non-intubated and intubated COVID-19 patients, respectively.

**Methods:**

We searched the MEDLINE, EMBASE, and Cochrane databases, as well as one Korean domestic database, on July 9, 2021, and updated the search 9 times to September 14, 2022. Studies that compared prone and non-prone positions in patients with COVID-19 were eligible for inclusion. The primary outcomes were mortality, need for intubation, and adverse events.

**Results:**

Of the 1259 records identified, 9 randomized controlled trials (RCTs) and 23 nonrandomized studies (NRSs) were eligible. In the non-intubated patients, the prone position reduced the intubation rate compared with the non-prone position in 6 RCTs (*n* = 2156, RR 0.81, *P* = 0.0002) and in 18 NRSs (*n* = 3374, RR 0.65, *P* = 0.002). In the subgroup analysis according to the oxygen delivery method, the results were constant only in the HFNC or NIV subgroup. For mortality, RCTs reported no difference between prone and non-prone groups, but in NRSs, the prone position had a significant advantage in mortality [18 NRSs, *n* = 3361, relative risk (RR) 0.56, *P* < 0.00001] regardless of the oxygen delivery methods shown in the subgroup analysis. There was no RCT for intubated patients, and mortality did not differ between the prone and non-prone groups in NRSs. Adverse events reported in both the non-intubated and intubated groups were mild and similar between the prone and non-intubated groups.

**Conclusion:**

For non-intubated patients with COVID-19, prone positioning reduced the risk of intubation, particularly in patients requiring a high-flow oxygen system. However, the survival benefit was unclear between the prone and non-prone groups. There was insufficient evidence to support the beneficial effects of prone positioning in intubated patients.

*Trial registration* This study was registered in the Prospective Register of Systematic Reviews on February 16, 2022 (Registration No.: CRD42022311150).

**Supplementary Information:**

The online version contains supplementary material available at 10.1186/s40001-022-00953-z.

## Background

Pulmonary involvement is common in COVID-19 patients and approximately 10–20% of hospitalized patients with COVID-19 had severe respiratory failure requiring mechanical ventilation [1]. Interventions to reduce mortality risk have been actively attempted in COVID-19 patients with respiratory failure, and the prone position is one of them.

Randomized trials and meta-analysis supported that the prone position showed favorable outcomes, including improved oxygenation, respiratory mechanics, and survival in patients with moderate-to-severe non-COVID-19 acute respiratory distress syndrome (ARDS) [2–4]. Similarly, in patients with COVID-19, several studies reported that prone positioning showed improved oxygenation [5–7] and reduced mortality [8, 9]. However, patients included in those studies varied in severity and degree of oxygen requirement, from nasal prong to mechanical ventilation. In particular, it is interesting that the awake-prone position was applied in many COVID-19 patients who were not critically ill, but had an oxygen demand and the possibility of respiratory failure. A recent meta-analysis showed that an awake-prone position reduced the risk of intubation, especially in COVID-19 patients requiring advanced respiratory support [10]. However, the result was mainly driven by one large trial, and two additional large randomized trials have been published recently. They have shown conflicting results regarding the effectiveness of prone position in patients with high-flow oxygen therapy or non-invasive ventilation [11, 12].

For intubated patients, relatively fewer studies had been performed compared to those for non-intubated patients and most studies compared oxygenation status before and after the application of prone position. Since prone positioning was considered to be performed in severe respiratory failure patients if possible, few observational studies compared outcomes of patients with and without prone position. However, the effect of prone position on mortality was inconsistent between studies [9, 13] and there has been no meta-analysis or systemic review of these comparisons.

Therefore, this study aimed to evaluate the efficacy and safety of the prone position in COVID-19 patients with respiratory failure and to analyze which prone position could be recommended among non-intubated and intubated patients, respectively.

## Methods

This study followed the recommendations outlined in the Preferred Reporting Items for Systematic Reviews and Meta-Analyses (PRISMA) 2020 guidelines [14] (Additional file 1). This study was registered in the Prospective Register of Systematic Reviews (PROSPERO) on February 16, 2022 (registration number CRD42022311150).

### Eligibility criteria

The inclusion criteria were as follows: (1) population—studies targeting patients with moderate-to-severe COVID-19; (2) intervention and comparator—studies comparing prone position to non-prone position; (3) outcomes—studies reporting the clinical outcomes including mortality, need for invasive mechanical ventilation, adverse events; (4) studies published after 2020; (5) study designs—randomized clinical trials (RCTs) or nonrandomized studies (NRSs) with a comparator group; and (6) full-text articles in English or Korean language. The exclusion criteria were as follows: (1) studies that did not target patients with confirmed COVID-19; (2) studies that did not compare the prone position to the non-prone position; (3) studies that did not report our outcomes of interest; and (4) duplicated studies.

### Information sources and search strategy

We searched the following electronic databases: international databases (Ovid MEDLINE, Ovid EMBASE, the Cochrane Central Register of Controlled Trials), and the Korean domestic database (KMBASE) on July 9, 2021. Since new evidence on the prone position of COVID-19 patients is continuously produced, we updated the search 9 times from September 10, 2021, to September 14, 2022. We searched Ovid-MEDLINE for updates and reference lists of previously published reviews. We used Boolean operators such as (2019-nCoV OR COVID-19 OR Wuhan) AND (prone position OR prone posture OR proning). The search strategy is presented in Additional file 2.

### Selection process

Four authors (HJL, JoK, JP, and JuK) independently screened the retrieved citations by title and abstract in COVIDENCE (https://www.covidence.org/) according to the inclusion and exclusion criteria. Full texts were assessed for the final decision of inclusion or exclusion by two authors (HJL and JoK). If an agreement was not reached between the two authors, it was reached through discussion with the third author (MC).

### Data items and extraction

The following data were extracted from the eligible studies using an electronic spreadsheet (Microsoft Excel) of data abstraction form: first author, published year, study design and setting, study location, sample size in each arm, oxygen therapy method, prone position protocol and duration, and outcomes of interest. Two authors (JP and JuK) extracted information from each included study, and two other authors (WIC and JJ) checked the data independently.

### Study outcomes

The primary outcomes were mortality, the need for intubation (in the case of non-intubated patients), and adverse events. The secondary outcomes were the length of stay (LOS) in the hospital or intensive care unit (ICU), ICU-free days, and ventilator-free days.

### Study risk-of-bias assessment

A validated tool was used according to the study design to evaluate the risk of bias in the included studies. The Cochrane risk-of-bias tool (RoB) 1.0 [15] was used for RCTs, and the Risk of Bias Assessment tool for Nonrandomized Studies (RoBANS) 2.0 [16] which was updated from RoBANS 1.0 [17] for nonrandomized studies. Two independent authors (WIC and JJ) conducted quality assessments of the studies, and disagreements were resolved by a third author (MC).

### Effect measures and synthesis methods

Based on the data extraction results, the meta-analysis was performed as follows. Relative risks (RR) with 95% confidence intervals (CI) for discrete outcome data and mean differences (MD) with 95% CI for continuous outcome data were calculated using the random-effects model because of heterogeneity across studies. Statistical significance was set at *P* < 0.05. To assess between-study heterogeneity, we displayed forest plots and calculated *I*^2^ statistics with a value of > 75%, considered high heterogeneity [18]. A subgroup analysis was performed based on oxygen delivery methods [conventional oxygen therapy (COT), high-flow nasal cannula (HFNC), non-invasive ventilation (NIV), or invasive mechanical ventilation (IMV)]. When more than one oxygen delivery method was used, the studies were classified based on the method by which the majority of patients received oxygen. To assess publication bias, we generated funnel plots for the primary outcomes reported in at least ten studies and performed Egger's linear regression test. We used Review Manager (RevMan) 5.4 [19] to synthesize the data and R version 4.2.1 [20] for Egger's linear regression test.

### Certainty of evidence assessment

We used the Grading of Recommendations, Assessment, Development, and Evaluation (GRADE) [21] to assess the certainty of the evidence of the primary outcomes. Two authors (WIC and JJ) assessed the certainty of the evidence as high, moderate, low, or very low, and discrepancies were resolved by a third author (MC).

## Results

### Study selection

**Fig. 1:**
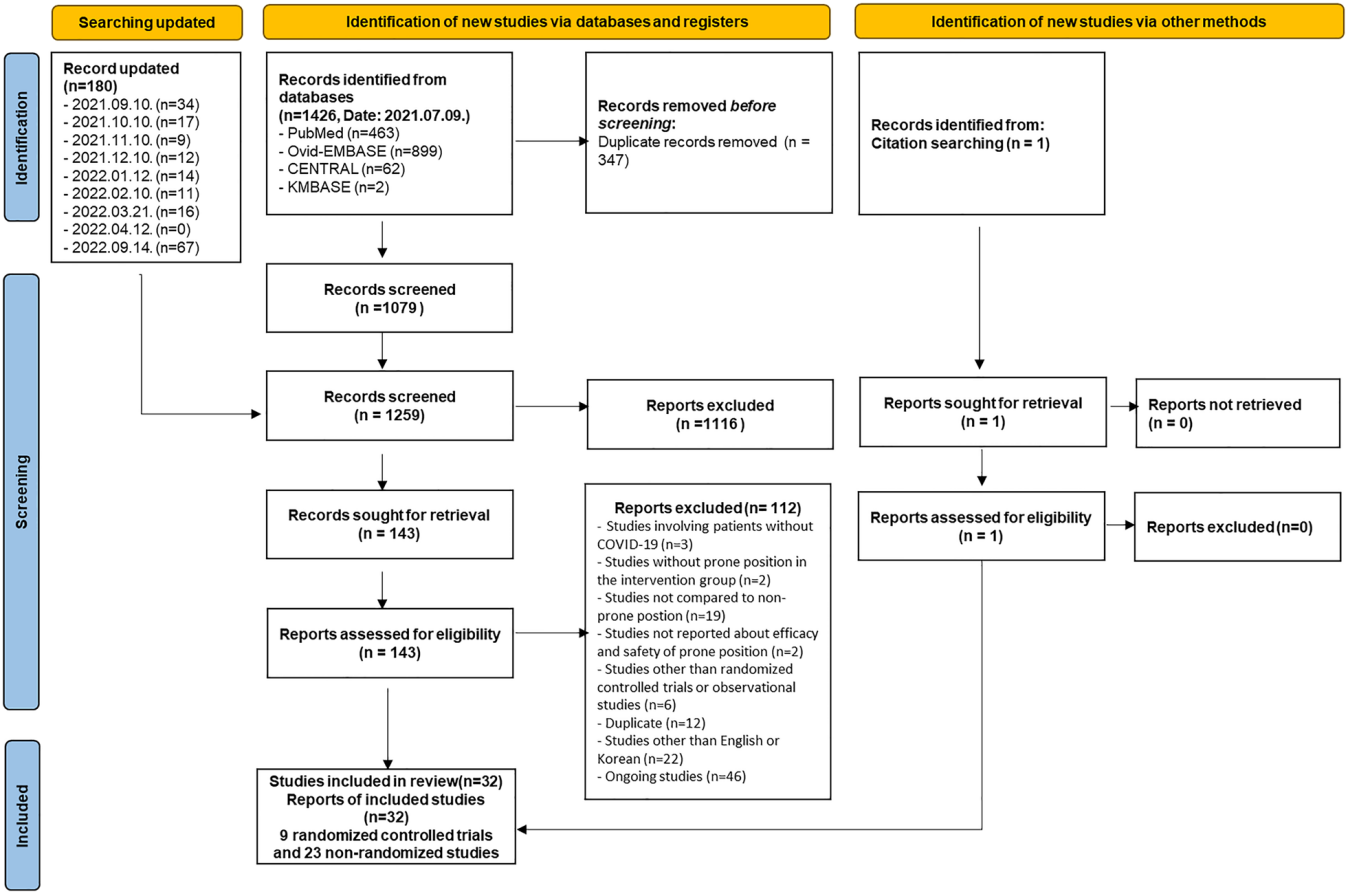
PRISMA flowchart

The study selection process is illustrated in Fig. 1. A total of 1426 records were identified using the search strategy on July 9, 2021, and 347 duplicate records were removed before the screening. One hundred and eighty records were updated until September 14, 2022. Of the 1259 records, 1116 were excluded after screening using titles and abstracts. Subsequently, the full texts of the 143 reports were retrieved. After reviewing the eligibility of the original texts, 9 RCTs with 2431 patients (sample size range, 27–1121) [11, 12, 22–28] and 23 nonrandomized studies (NRSs) including 2 nonrandomized controlled trains with 744 patients (sample size range, 243–501) [29, 30], 7 prospective cohort studies with 761 patients (sample size range, 32–335) [5, 7, 31–35], and 14 retrospective cohort studies with 3119 patients (sample size range, 20–827) [6, 8, 9, 36–46] were included in our review. The list of excluded studies and reasons for exclusion are presented (Additional file 3).

The characteristics of the included studies are summarized in Table 1. Eleven studies originated in Europe [6, 8, 26, 27, 30, 32, 33, 37, 44–46], eight from Asia [5, 25, 34–36, 38, 40, 41], five from North America [9, 23, 28, 29, 39], four from the South America [11, 31, 42, 43], two from Africa [7, 24], and two from multiple countries [12, 22]. Seventeen studies [6, 8, 11, 12, 22, 23, 25, 27, 29, 31, 32, 34, 41, 43–46] were conducted at multiple centers and others at single centers. In most of the studies, including the all RCTs, patients were provided through the COT, HFNC, or NIV, and in three NRSs studies [9, 38, 45], through mechanical ventilation. The proning protocols varied in terms of time and frequency of sessions, such as at least 2–18 h per day or no restrictions in time and frequency. The reported proning durations varied. The average proning time per day (3–15 h per day) [11, 22, 26, 27, 29–31, 34, 40, 45], the total number of proning session (2–4 sessions) [11, 30, 40], or days in proning (2.5–13 days) [11, 12, 27, 30, 31, 40, 44, 45] were reported.

**Table 1:** The basic characteristics of studies included in this review

First author, published year	Study design	Study setting	Study location	Enrollment period	Total sample size (prone/non-prone)	Age (year, IQR or SD) (prone/non-prone)	Male (%) (prone/non-prone)	Oxygen therapy methods	Proning protocol	Location of proning	Proning duration in prone group	
											Daily	Total
Ehrmann [22]	RCT	Multicenter	Canada, France, Ireland, Mexico, USA, Spain	2020.4.2.–2021.1.26	1121 (564/557)	61.5 (13.3)/60.7 (14.0)	67/66	HFNC	As long and as frequently as possible each day	ICU, Ward, ER	5 (IQR 1.6–8.8) h	NR
Jayakumar [25]	RCT	Multicenter	India	NR	60 (30/30)	54.8 (11.1)/57.3 (12.1)	83.3/83.3	Nasal prongs, face mask, non-rebreather mask, HFNC, or NIV	≥ 6 h/day (cumulative)	ICU	≥ 6 h in 43% of intervention group	NR
Kharat [26]	RCT	Single center	Switzerland	2020.4.6.–2020.4.25	27 (10/17)	54 (14)/60 (11)	60/65	Nasal cannula	≤ 12 h/day	Ward	295 (SD 216) m	NR
Rosén [27]	RCT	Multicenter	Sweden	2020.10.7.–2021.2.7	75 (35/39)	66 (53–74)/65 (55–70)	64/82	HFNC, NIV	≥ 16 h/day	Ward, ICU	9.0 (IQR 4.4–10.6) h	4.2 (1.7–5.7) d
Taylor [28]	RCT	Single center	US	2020.6.1.–2020.8.31	40 (27/13)	56 (45–66)/60 (54–63)	63/77	Nasal cannula, MFNC	As long as possible and allowed to return to the supine position as necessary	Ward	NR	NR
Fralick [23]	RCT	Multicenter	Canada, US	2021.3.-2021.5	248 (126/122)	59.5 (45–68)/54 (44–62)	65/63	Nasal cannula, mask, HFNC	Four sessions/day (up to 2 h/sessions), encouraged to sleep in pone position overnight	Ward	NR	From randomization to first 72 h: 6 (1.5–12.8) h, from 72 h to 7 d: 0 (0–12) h
Gad [24]	RCT	Single center	Egypt	2020.6.-2020.9	30 (15/15)	49.0 (38–62)/46.0 (33–51)	60.0/53.3	High flow with non-rebreathing facemask	Each session for 1–2 h according to patient to tolerability with 3 h apart during waking hours	Critical care isolation	NR	NR
Ibarra-Estrada [11]	RCT	Multicenter	Mexico	2020.5.5.–2021.1.26	430 (216/214)	58.6 (15.8)/58.2 (15.8)	61.1/58.9	HFNC	As long as possible	Intermediate or intensive care unit	4 sessions/d (3–5) 3.4 (3–3.6) h/session	9.4 (5.6–12.9) h for 6 (3.7–9) ds
Alhazzani [12]	RCT	Multicenter	Multicountry	2020.5.19–2021.5.13	400 (205/195)	56.8 (12.5)/58.3 (13.2)	73/69	HFNC, LFNC, NPPV	8 h/d–10 h/d with 2 to 3 breaks (1–2 h each), if needed	ICU or a monitored acute care unit	NR	3 (1–5) d
Musso [30]	NRCT	Single center	Italy	Int 2020.12.16.–2021.5.30. Cont 2020.4.1.–2020.12.15	243 (81/162)	68 (60–75)/69 (61–78)	76/72	NIV	As long as possible, at least 1 session/day lasting ≥ 8 h scheduled overnight	Subintensive care unit	12.2 (10.1–13.8) h, 2 session/d	6 (5–8) d
Qian [29]	NRCT	Multicenter	US	2020.5.13.–2020.12.11	501 (243/258)	61.6 (15.4)/60.3 (15.2)	56.6/56.8	HFNC, LFNC, NIV	Encouraged as often and consistently as possible	NR	4.2 (1.8–6.7) h	NR
Ferrando [32]	Prospective cohort study	Multicenter	Spain, Andorra	2020.3.12.–2020.6.9	199 (55/144)	60.0 (54.0–70.0)/63.0 (55.0–71.0)	75.9/72.7	HFNC	> 16 h regardless of the number of sessions	ICU	NR	NR
Ni [5]	Prospective cohort study	Single center	China	2020.1.31.–2020.2.15	52 (17/35)	60 (12)/64 (12)	64.7/60	NR	≥ 4 h/day for 10 days	Provisional ICU	NR	NR
Zang [35]	Prospective cohort study	Single center	China	2020.2.1.–2020.4.30	60 (23/37)	63 (59–71)/66 (60–72)	56.5/70.3	O_2_ storage mask	1–2 h/session, 3–4 sessions/day for more than 5 consecutive days	NR	NR	13.4 (SD 8.0) h
Bahloul [7]	Prospective cohort study	Single center	Tunisia	2020.9.1.–2020.12.4	38 (21/17)	61 (10)/60 (12)	76.2/NR	Facial mask, HFNC	2–4 h followed by 2 h of supine positioning during the day, and to sleep in a proning at night, when possible	ICU	NR	NR
Esperatti [31]	Prospective cohort study	Multicenter	Argentina	2020.6.–2021.1	335 (187/148)	57 (47–66)/66.5 (56.5–75)	76/72	HFNC	≥ 6 h/day, no time limits for prone position	ICU	12 (IQR 9–16) h	5 (IQR 3–8) d
Sryma [34]	Prospective cohort study	NR	India	NR	45 (30/15)	50.9 (10.1)/57.5 (12.2)	96.7/60	NIV, HFNC, COT	≥ 2 h/session, target duration of 8 h/day	NR	7.5 (range 4–12) h on the first day	NR
Pierucci [33]	Prospective cohort study	Single center	Italy	2020.3.11.–2020.4.30	32 (16/16)	59 (11)/70 (15)	81/62	Int: spontaneously breathing Cont: HFNC, CPAP, NIV	As long as possible with intervals for meals and other personal care			
Jagan [39]	Retrospective cohort study	Single center	US	2020.3.24.–2020.5.5	105 (40/65)	56.0 (14.4)/65.8 (16.3)	50/56.9	Non-intubation	≥ 1 h/day for at least 5 sessions, ≥ 1 h/overnight	NR	NR	NR
Padrão [42]	Retrospective cohort study	Single center	Brazil	2020.3.1.–2020.4.30	166 (57/109)	51.8 (13)/61.4 (13.6)	70/66	Nasal cannula, venturi mask, non-rebreather mask	≥ 4 h in the first session, stimulated twice daily to maintain proning	NR	First sessio*n* < 1 h 6%, 1–2 h 14%, 2–3 h 12%, 3–4 h 10%, > 4 h 58%	NR
Barker [37]	Retrospective cohort study	Single center	UK	2020.3.26.–2020.6.26	20 (10/10)	59 (55–63)/65 (55–71)	60/60	NIV (Int 90%, Con 70%)	30 m–2 h, repeated as many times as comfortable	ICU	NR	120 (IQR 76–161) m, 4 (IQR 1–7) sessions/patient
Jouffroy [6]	Retrospective cohort study	Multicenter	France	2020.2.20.–2020.4.24	379 (40/339)	59.5 (56–64)/62 (53–69)	90/75.2	HFNC	3–6 h/session, twice a day	ICU	NR	2.5 (IQR 1.6–3.4) d, 3 (IQR 2–5) sessions
Loureiro-Amigo [8]	Retrospective cohort study (SEMI-COVID-19)	Multicenter	Spain	2020.3.1.–2020.8.31	163 (60/103)	66.57 [59.2–72.4]/70.81 [60.6–74.2]	71.7/68.9	Venturi masks, rebreathing masks	NR	Ward	NR	NR
Prud’homme [44]	Retrospective cohort study	Multicenter	France	2020.3.20–2020.4.20	96 (48/48)	62 (11)/61 (18)	77.1/64.6	COT, HFNC	≥ 3 h/day during 3 consecutive days, 1 to 12 h/session	Non-ICU	3–8 h in 67%, > 5 h in 38% of intervention group	6.9 (SD 5.2) d
Shelhamer [9]	Retrospective cohort study	Single center	US	2020.3.25.–2020.5.2	261 (62/199)	60.0 (54.3–66.5)/66.0 (55.0–74.5)	67.7/60.3	IMV	≥ 16 h in the afternoon, supine position the following morning	Traditional ICU, Converted floor ICU	NR	NR
Stilma [45]	Retrospective cohort study	Multicenter	Netherlands	2020.3.1.–2020.6.1	734 (438/296)	Without indication for proning 65.0 (10.3)/64.2 (11.4) With indication for proning 62.6 (11.2)/66.6 (9.1)	72.6/73.3	IMV	NR	ICU	15.0 (IQR 10.5–21.0) h	3 (2–3) d
Tonelli [46]	Retrospective cohort study	Multicenter	Italy	2020.3.1.–2020.6.1	114 (38/76)	61 (32 − 75)/70 (33 − 80)	66/73	HFNC, CPAP, NIV	≥ 3 h before back to supine	ICU	NR	NR
Perez-Nieto [43]	Retrospective cohort study (APRONOX study)	Multicenter	Mexico, Ecuador	2020.5.1.–2020.6.12	827 (505/322)	53.4 (13.9)/55.8 (14.5)	73.3/71.4	LFNC, HFNC or a non-rebreather mask	≥ 2 h continuously	ICU 13% non-ICU 87%	NR	12 (IQR 8–24) h
Koike [40]	Retrospective study	Single center	Japan	Int: 2020.10.1.–2020.12.1. Cont: 2020.12.1.–2020.3.31	58 (27/31)	71 (55–77)/63 (49–70)	90/87	Simple O_2_, HFNC, NPPV	Discontinued if the patient developed intolerable respiratory distress, tachypnea > 35 bpm, or new unacceptable back pain during proning	ICU	2 (2–3) sessions/d 180 (120–240) m	13 (7–16) d
Altinay [36]	Retrospective study	Single center	Turkey	2020.3.15.–2020.6.15	48 (25/23)	62.4 (10.9)/72.6 (10.1)	44/39.1	Nonrebreather mask oxygen	18 h intermittently in a day	ICU	NR	NR
Numata [41]	Retrospective study	Multicenter	Japan	2020.7–2021.2	108 (54/54)	68 (58–76)/70 (59–79)	68.5/42.6	HFNC	As long as possible, at least 3 times a day and for at least 6 h per day	Severe COVID-19 patient unit	NR	NR
Chen [38]	Retrospective study	Single center	China	2020.1.9.–2020.4.10	40 (17/23)	69 (56–87)/72 (54–89)	64.7/78.3	IMV	NR	ICU	NR	NR

RCT, randomized controlled trial; NRCT, nonrandomized controlled trial; HFNC, high-flow nasal cannula; MFNC, medium-flow nasal cannula; ICU, intensive care unit; ER, emergency room; IQR, interquartile range; SD, standard definition; NR, not reported; NIV, non-invasive ventilation; COT, conventional oxygen therapy; ECMO, extracorporeal membrane oxygenation; IMV, invasive mechanical ventilation; CPAP, continuous positive airway pressure; LFNC, low-flow nasal cannula; NPPV, non-invasive positive pressure ventilation

### Risk of bias in studies

The majority of RCTs were assessed as having a low risk of bias in all the dimensions. In more than half of the NRSs, the domains of the possibility of target group comparison and selection were rated as having a high risk of bias (Additional file 4: Fig. S1). However, serious problems did not occur because the domains of exposure measurement, blinding of assessors, outcome assessment, and selective outcome reporting were assessed as having a low risk of bias in most NRSs.

### Non-intubated group

#### Mortality

**Fig. 2:**
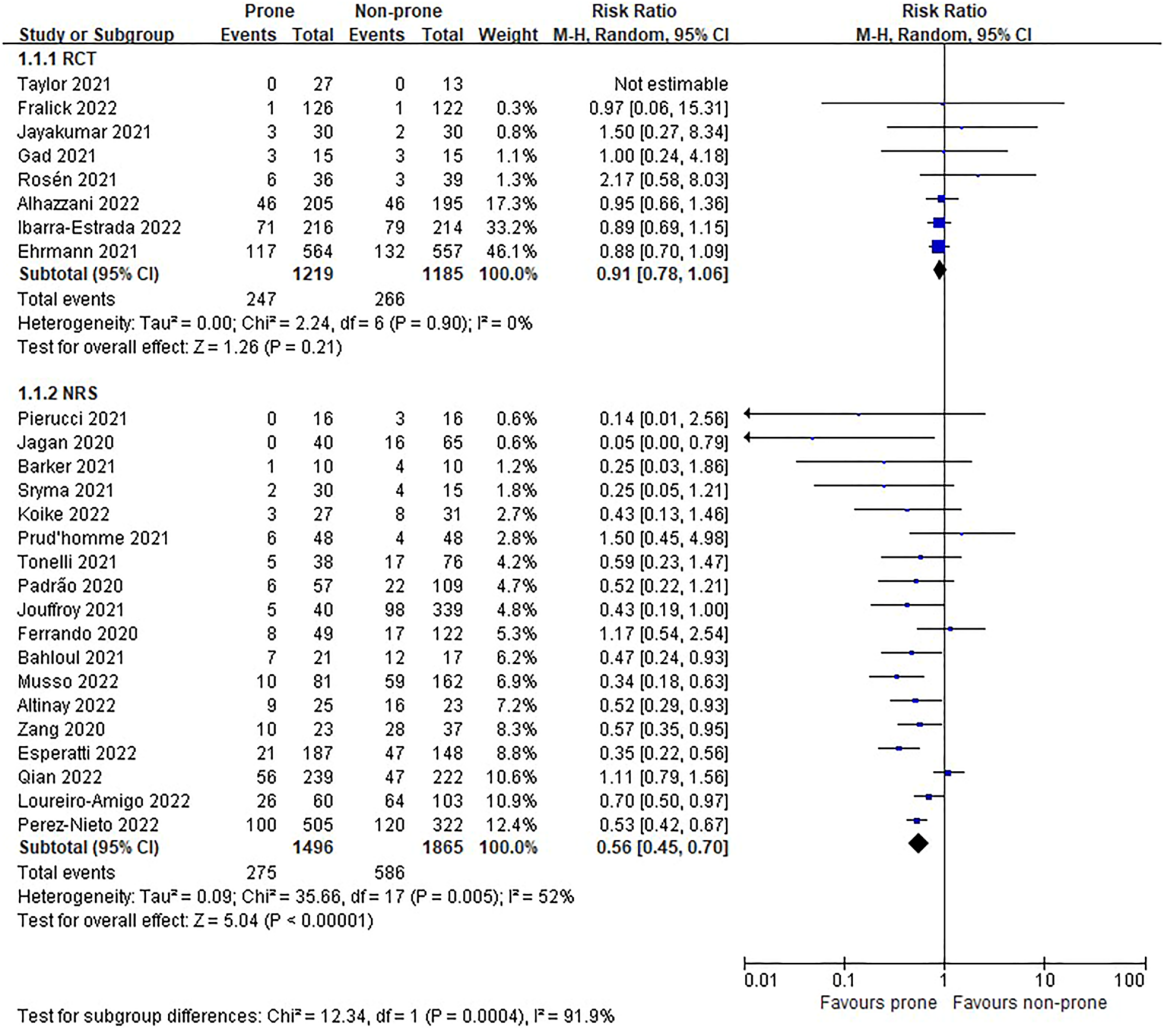
Mortality of non-intubated patients

**Fig. 3:**
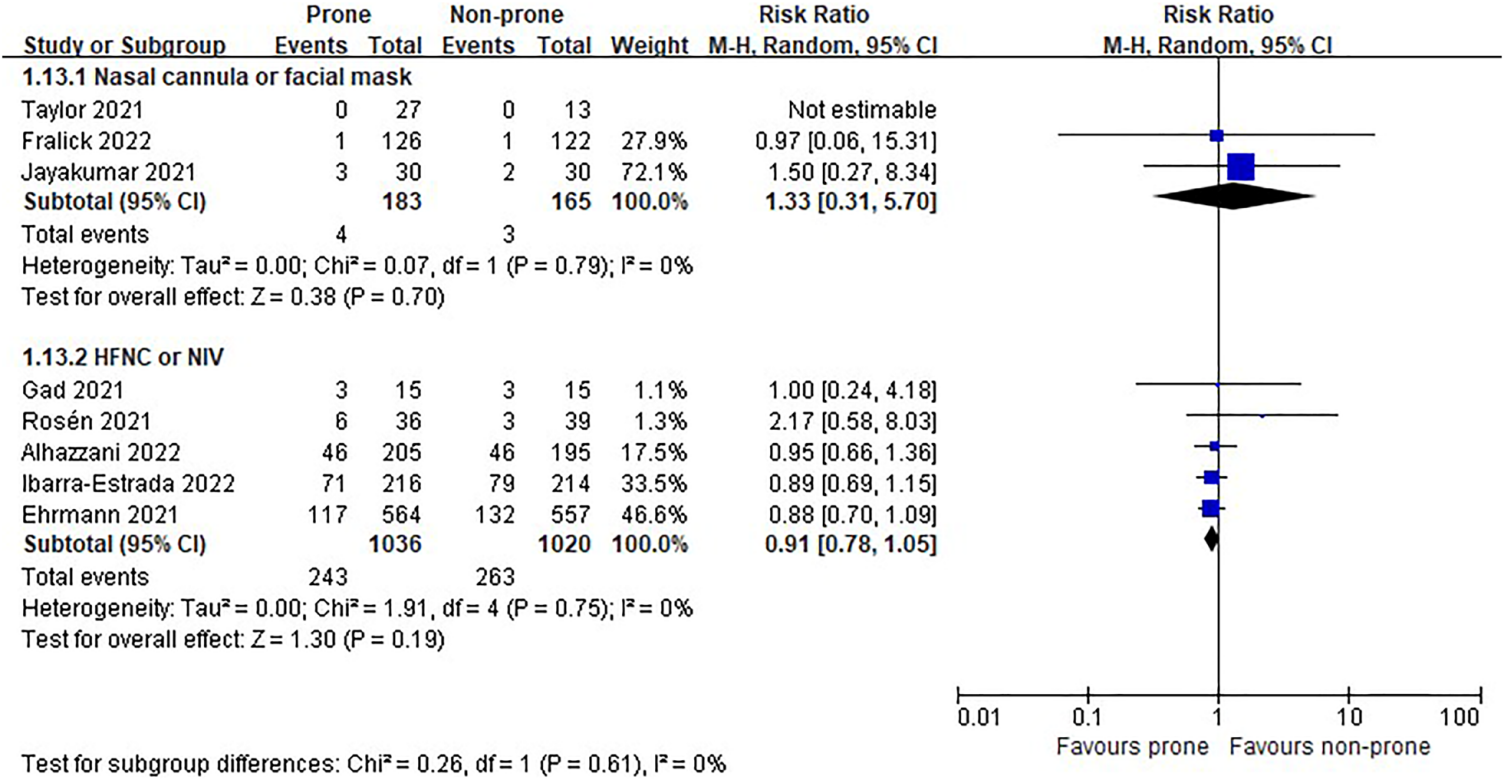
Subgroup analysis of mortality by oxygen delivery methods in non-intubated patients of randomized studies

**Fig. 4:**
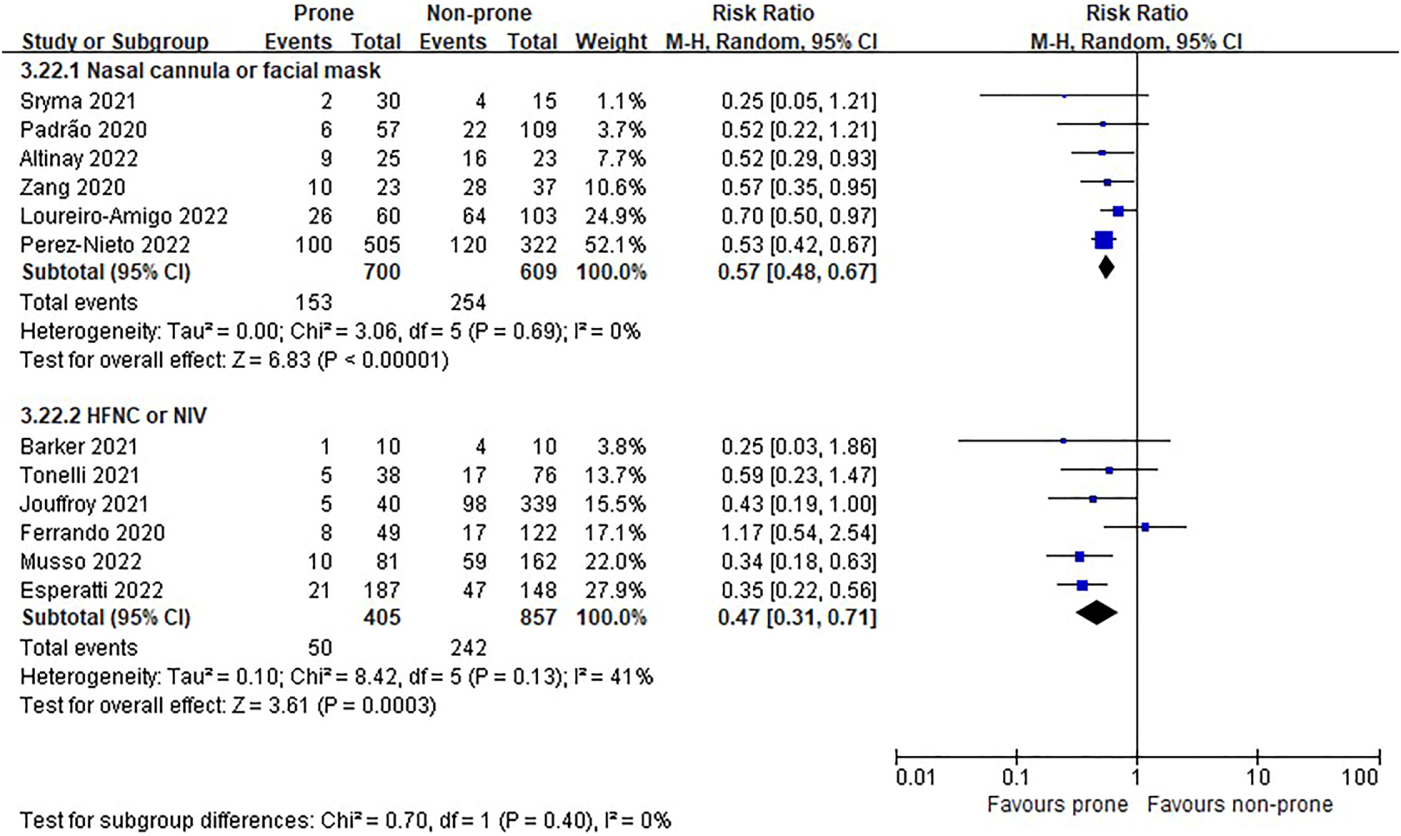
Subgroup analysis of mortality by oxygen delivery methods in non-intubated patients of non-randomized studies

In the eight RCTs [11, 12, 22–25, 27, 28], there was no difference in morality between prone and non-prone groups (high certainty of evidence), but in the NRSs [6–8, 29–37, 39, 40, 42–44, 46], the prone position had a significant advantage of survival in the non-intubated patient group (18 NRSs, *n* = 3361, RR 0.56, 95% CI 0.45 to 0.70, *P* < 0.00001, *I*^2^ = 52%, very low certainty of evidence; Fig. 2). The subgroup analysis for the oxygen delivery method showed constant results (Figs. 3, 4). In NRSs, prone reduced mortality compared to non-prone in the nasal cannula or facial mask group (6 NRSs, *n* = 1309, RR 0.57, 95% CI 0.48–0.67, *P* < 0.00001, *I*^2^ = 0%) and the HFNC or NIV group (6 NRSs, *n* = 1262, RR 0.47, 95% CI 0.31–0.71, *P* = 0.0003, *I*^2^ = 41%). Although the funnel plot for mortality in NRSs was asymmetric, we observed no evidence of publication bias in Egger's linear regression test (*P* = 0.2192, Additional file 4: Fig. S2).

#### Need for intubation

**Fig. 5:**
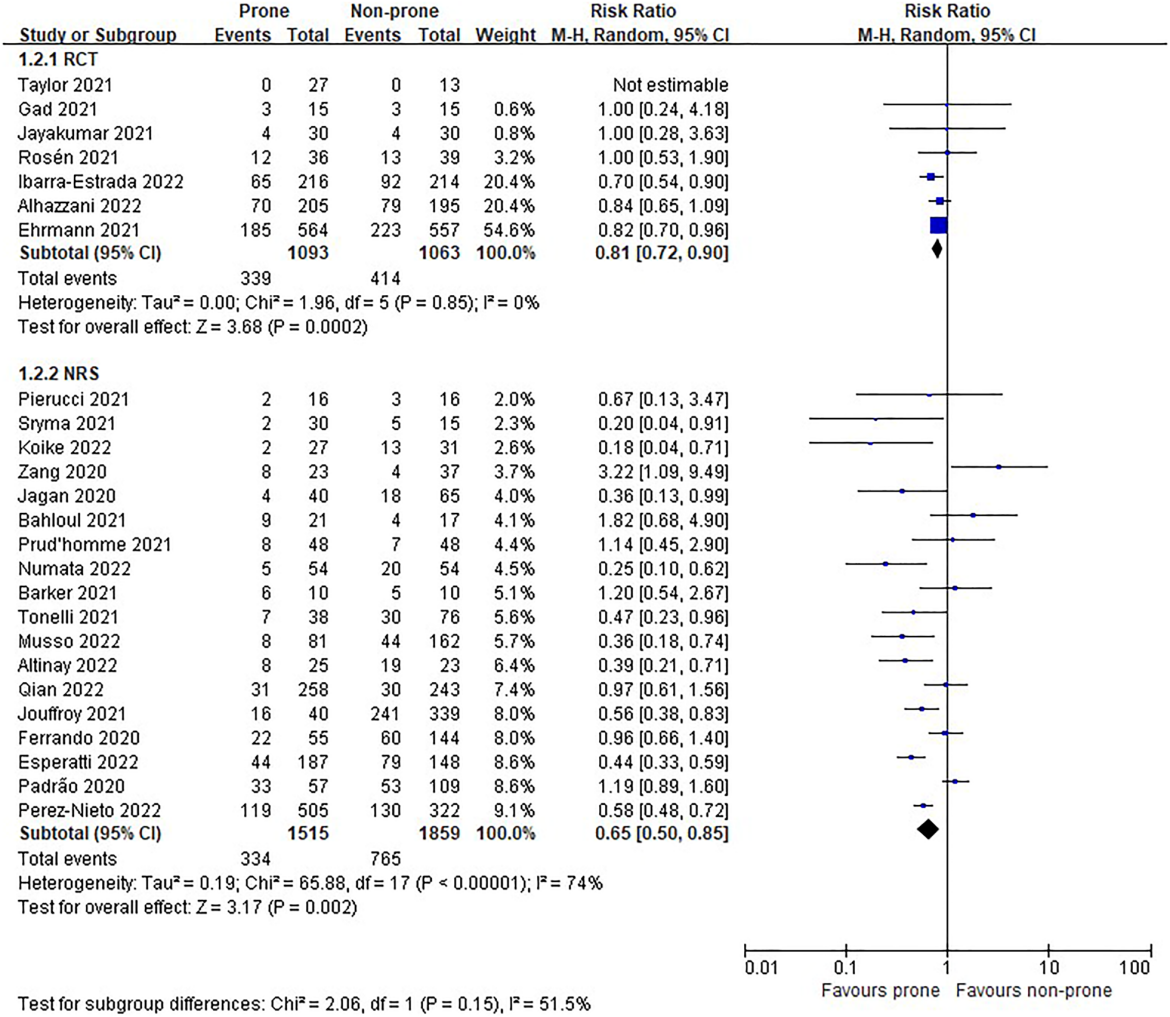
Need for intubation of non-intubated patients

**Fig. 6:**
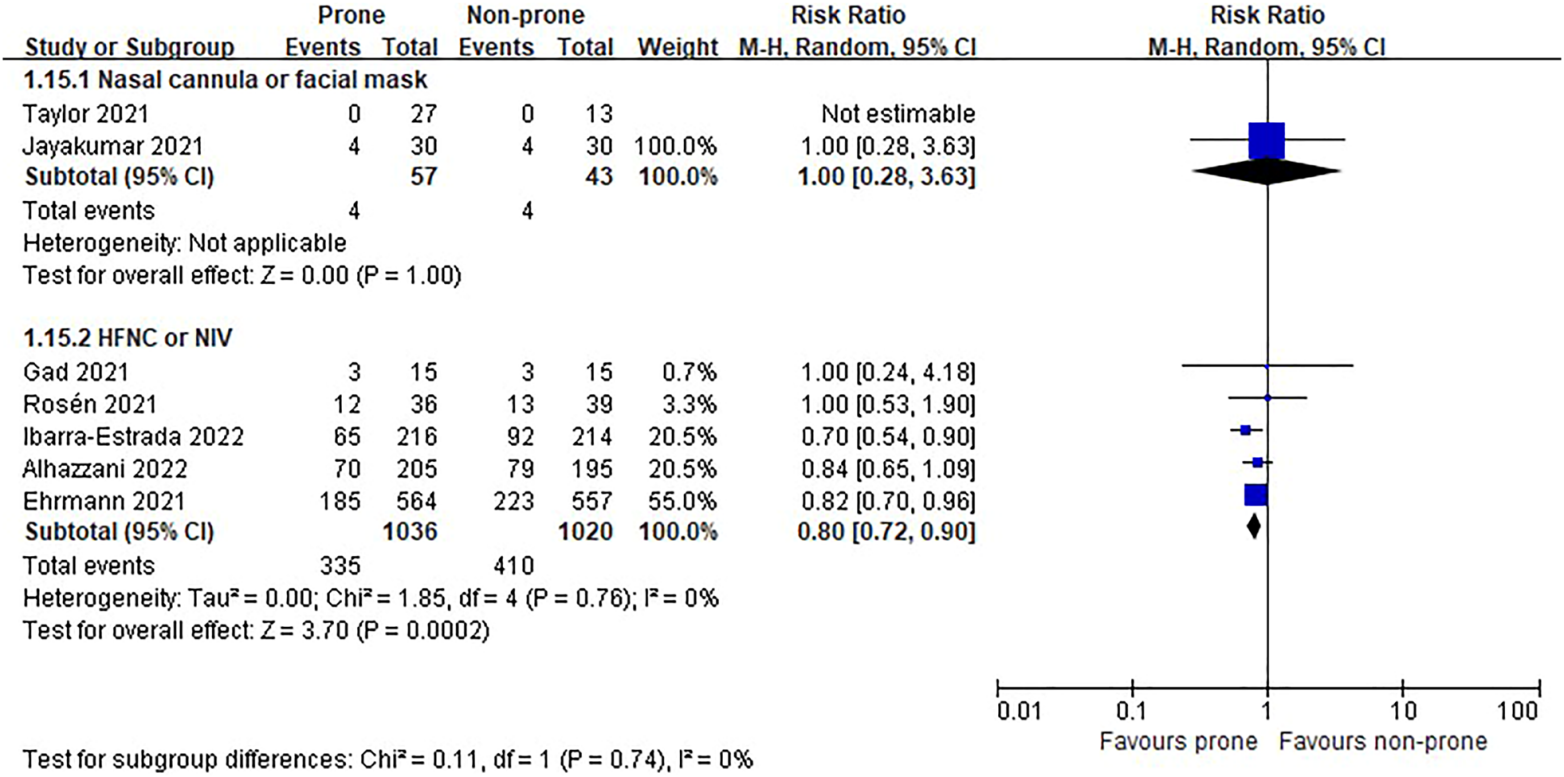
Subgroup analysis of intubation rate by oxygen delivery methods in non-intubated patients of randomized controlled trials

**Fig. 7:**
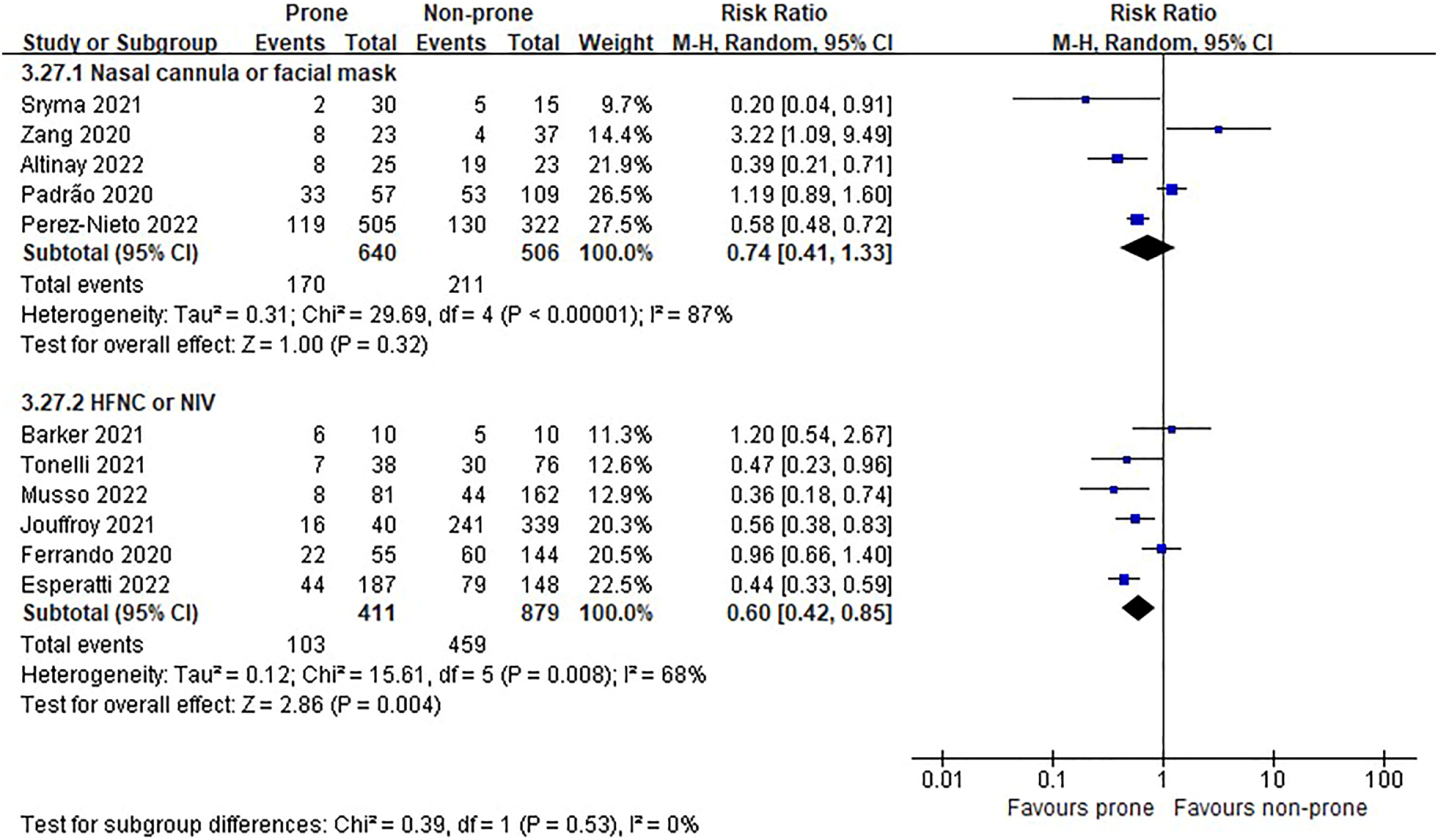
Subgroup analysis of intubation rate by oxygen delivery methods in non-intubated patients of non-randomized studies

The intubation rate of the prone group was significantly lower than that of the non-prone group in 7 RCTs (*n* = 2156, RR 0.81, 95% CI 0.72 to 0.90, *P* = 0.0002, *I*^2^ = 0%, high certainty of evidence) [11, 12, 22, 24, 25, 27, 28] and 18 NRSs (*n* = 3374, RR 0.65, 95% CI 0.50 to 0.85, *P* = 0.002, *I*^2^ = 74%, very low certainty of evidence) [6, 29, 30, 36, 37, 39–44, 46] (Fig. 5). In the subgroup analysis according to the oxygen delivery method, proning showed advantage only in the HFNC or NIV subgroup (Figs. 6, 7). Although the funnel plot for the intubation rate of nonrandomized studies was asymmetric, we observed no evidence of publication bias in Egger's linear regression test (*P* = 0.8453, Additional file 4: Fig. S3).

#### Adverse events

Seven RCTs reported adverse events [11, 12, 22, 25–28]. The incidence of cardiac arrest (at any time) was similar between the prone and non-prone positions (prone vs. non-prone 3/564 vs. 1/557, p value not reported) [22] and skin breakdown and vomiting were also similar between the two groups (moderate certainty of evidence, Additional file 4: Fig. S4). Six NRSs reported adverse events in the prone group [5, 30, 34, 42, 44, 46], which were mainly mild (very low certainty of evidence, Additional file 4: Table S1).

#### Length of stay in hospital or ICU

Length of stays in hospital or ICU were not different between prone and non-prone groups in both RCTs and NRSs (Additional file 4: Figs. S5, S6).

#### ICU-free days and ventilator-free days

ICU-free days were not different in RCTs, and ventilator-free days were not different in RCTs and NRSs between prone and non-prone groups (Additional file 4: Figs. S7, S8).

### Intubated group

**Fig. 8:**
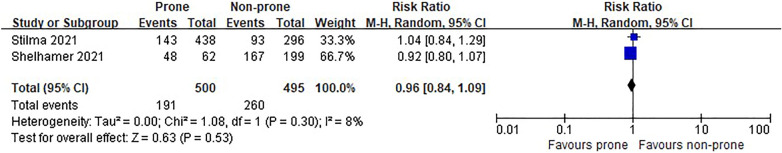
Mortality of intubated patients in non-randomized studies

Only NRSs included intubated patients [9, 38, 45]. Mortality did not differ between the prone and non-prone groups in 2 NRSs [9, 45] (Fig. 8), but 1 NRS [38] reported better survival in prone group than in non-prone group (adjusted hazard ratio 0.282, 95% CI 0.126 to 0.63) (very low certainty of evidence). Incident occurrence of peripheral line removal in two patients during positioning was reported in 1 NRS [9] (very low certainty of evidence, Additional file 4: Table S1). Hospital LOS and ICU LOS were longer in prone group than non-prone group (hospital LOS, one study [9], *n* = 261, MD 10.1 days, 95% CI 7.39 to 12.81 days, *P* < 0.00001; ICU LOS, one study [45], *n* = 734, MD 2.71 days, 95% CI 0.77 to 4.65 days, *P* = 0.006). Ventilator-free days did not differ between the prone and non-prone groups in 2 NRSs [9, 45] (Additional file 4: Fig. S9), and no studies reported ICU-free days between prone and non-prone groups.

The GRADE summary of findings table of primary outcomes is reported in Table 2.

**Table 2:** GRADE summary of findings table of primary outcomes

Outcomes	Study design	Anticipated absolute effects^c^ (95% CI)		Relative effect (95% CI)	№. of participants (studies)	Certainty of the evidence (GRADE)
		Risk with non-prone	Risk with prone			
*Non-intubated patients*						
Mortality	RCT	224 per 1000	204 per 1000 (175–238)	RR 0.91 (0.78–1.06)	2404 (8)	⨁⨁⨁⨁ High
	NRS	314 per 1000	176 per 1000 (141–220)	RR 0.56 (0.45–0.70)	3361 (18)	⨁◯◯◯ Very low^a^
Need for intubation	RCT	389 per 1000	315 per 1000 (280–351)	RR 0.81 (0.72–0.90)	2156 (7)	⨁⨁⨁⨁ High
	NRS	412 per 1000	267 per 1000 (206–350)	RR 0.65 (0.50–0.85)	3374 (18)	⨁◯◯◯ Very low^a^
Adverse events	RCT	24 per 1000	23 per 1000 (16–34)	RR 0.97 (0.66–1.43)	7011 (6)	⨁⨁⨁◯ Moderate^b^
	NRS	Cases in the prone group were reported as follows: desaturation or hemodynamic worsening 0/30 [34], back pain 2/30 [34] and 3/57 [42], bloating sensation 2/30 [34], gastric distension and vomit 0/81 [30], peripheral line removal 2/57 [42] and 2/81 [30], nasal skin ulceration 2/81 [30], major adverse events 0/48 [44], overall adverse events 0/17 [5] and 0/38 [46]			716 (6)	⨁◯◯◯ Very low^a,b^
*Intubated patients*						
Mortality	NRS	525 per 1000	504 per 1000 (441–573)	RR 0.96 (0.84–1.09)	995 (2)	⨁◯◯◯ Very low^a^
		Chen et al. [38] reported better survival in prone group than in non-prone group (adjusted hazard ratio 0.282, 95% CI 0.126 to 0.63)			40 (1)	
Adverse events	NRS	Cases in the prone group were reported as follows: endotracheal tube dislocation 0/62 [9], peripheral line removal 2/62 [9]			261 (1)	⨁◯◯◯ Very low^a,b^

GRADE Working Group grades of evidence

High certainty: we are very confident that the true effect lies close to that of the estimate of the effect

Moderate certainty: we are moderately confident in the effect estimate: the true effect is likely to be close to the estimate of the effect, but there is a possibility that it is substantially different

Low certainty: our confidence in the effect estimate is limited: the true effect may be substantially different from the estimate of the effect

Very low certainty: we have very little confidence in the effect estimate: the true effect is likely to be substantially different from the estimate of effect

CI, confidence interval; RR, risk ratio; RCT, randomized controlled trial; NRS, nonrandomized studies

^a^Downgrade for risk-of-bias concern in the domains of possibility of target group comparisons, target group selection, and confounder

^b^No more than 300 events

^c^The risk in the intervention group (and its 95% confidence interval) is based on the assumed risk in the comparison group and the relative effect of the intervention (and its 95% CI)

## Discussion

In this analysis, we divided patients with moderate-to-severe COVID-19 into intubated and non-intubated groups and investigated the benefit of the prone position. In summary, we found that prone position reduced the risk of intubation in non-intubated patients, particularly those supplied with high-flow oxygen systems. However, prone position did not reduce the risk of mortality in both the intubated or non-intubated groups. In non-intubated patients, survival benefit was only observed in observational studies, not in randomized trials. Moreover, there are no randomized controlled trials comparing prone to supine positions in intubated patients. Only a few observational cohort studies were included and did not show statistically better survival. In addition, ventilator-free days were significantly shorter in the prone position group. As a result, there is still insufficient evidence to support the beneficial effect of prone position in intubated patients.

However, it would be considered unethical to assign patients to the non-prone group since large randomized controlled trials and meta-analyses have already shown the beneficial effect of the prone position in patients with moderate and severe non-COVID ARDS [3, 47, 48]. In addition, although two different phenotypes of COVID-19 ARDS have been proposed, several studies have suggested similar clinical features between COVID-19 and non-COVID ARDS [49]. Compliance was higher in the COVID-19 initially, but decreased 3–7 days after onset with no difference from non-COVID-19 ARDS [50]. In addition, pathological characteristics and distribution of compliance were similar among studies of COVID-19 and non-COVID-19 ARDS [51, 52]. They also suggested that treatment previously considered for non-COVID-19 ARDS may apply to COVID-19 patients with respiratory failure [51]. In addition, observational cohort studies demonstrated that improved oxygenation and increased Pao_2_/Fio_2_ ratio after prone positioning even remained significantly higher after returning to the supine position [9, 53, 54]. Static lung compliance was also increased after prone positioning with reduced driving pressure [54]. In the analysis of lung computed tomography in COVID-19 ARDS, regional hyperinflation decreased, and inflation distribution was more homogenous in the prone position, which was also similar to other ARDS [55]. Based on previous experiences from non-COVID-19 ARDS and improvement of oxygenation in observational studies, guidelines recommended implementing the prone position in intubated patients with COVID-19. Further research is needed to evaluate the effect of prone position in intubated patients.

Meanwhile, studies on awake-prone positioning in non-intubated patients were conducted more actively, including randomized controlled trials. We included the most recently updated trials in this meta-analysis. Among non-intubated patients, the prone position group had a reduced risk of intubation. In the subgroup analysis according to oxygen delivery methods, prone positioning reduced intubation rates only in more severely ill patients receiving a high-flow oxygen system or non-invasive ventilation, and the risk of intubation was similar between the prone and non-prone groups in patients with low-flow oxygen. However, since there were only three randomized trials and a small number of patients included in the low-flow oxygen subgroup, it is insufficient to evaluate the effect of prone position. Therefore, more randomized trials will be needed. There was no difference in the mortality between prone and non-prone groups among randomized trials regardless of oxygen supply methods, which was consistent with previous meta-analysis [10]. There was also no significant difference in the length of ICU and hospital stays between the prone and non-prone groups.

Before COVID-19, there were only a few case series and retrospective observational studies about awake-prone positioning on acute respiratory failure [56–59]. Those studies showed that a prone position improved oxygenation, but a detailed investigation has not been done. Among non-intubated COVID-19 patients, improvement in oxygenation was also observed with prone positioning [5–7]. A physiologic study showed that dead space and shunt were reduced. As a result, V/Q mismatch was improved in the prone position, similar to the mechanism in the intubated patients [60]. Reduced intubation risk might be due to improvement of oxygenation and respiratory mechanics. Nevertheless, mortality rates were similar between the two groups in randomized trials. One suggestion why the mortality benefit was not achieved in the prone position group was low adherence to prone position in the awake-prone groups [37]. The duration of the awake-prone position depended on the patient’s effort, unlike when performed in intubated patients, who were usually sedated for prone position. In this analysis, durations were not stated in the studies and, if noted, varied with a median of 5 to 9 h. However, recent studies showed that a longer duration of prone position was associated with better outcomes [11, 22, 31], and Esperatti et al. suggested performing prone position for at least 8 h per day to reduce the risk of mortality [31]. Therefore, there is a possibility that the duration of prone positioning was not sufficient to achieve survival gain. However, other factors affecting the duration of prone position, such as poor medical conditions, also influence survival, so the effect of duration on mortality should be assessed more carefully.

Complications that may occur in the prone position include dislocation of the endotracheal tube and vascular lines, transient hypotension, vomiting, and pressure sores [61]. However, there were no serious adverse events such as unstable hemodynamics and removal of the endotracheal tube both in the intubated and non-intubated patients in our study. The incidence of minor complications was also similar between the two groups. The prone position can be a safe and effective intervention for patients with respiratory failure.

This study had several limitations. First, RCTs were limited to the non-intubated group. Therefore, there is a lack of evidence to evaluate the effect of the prone position in intubated patients. Second, the severity of the included patients varied within and among studies. The oxygen supply method in non-intubated patients was particularly heterogeneous, from the nasal cannula to the high-flow oxygen system and non-invasive ventilation. So we performed subgroup analysis by dividing the studies into two groups: low-flow and high-flow oxygen systems, and found that the reduced risk of intubation was observed only in more severe patients with high-flow oxygen systems or non-invasive ventilation. Third, most studies did not present the cycle and duration of prone positioning or were inconsistent, particularly for non-intubated patients. According to the previous guidelines, there were only recommendations for intubated patients to maintain a prone position for at least 16 h. Further studies to evaluate the effective duration of the prone position in non-intubated patients should be considered, although heterogeneous disease severities and oxygen requirements may make the investigation difficult.

## Conclusions

For non-intubated patients with COVID-19, prone positioning reduced the risk of intubation, particularly in patients requiring a high-flow oxygen system. However, the survival benefit was unclear between the prone and non-prone groups. There was insufficient evidence to support the beneficial effects of prone positioning in intubated patients, because only a few observational studies compared prone position and non-prone position. Further well-designed randomized controlled trials will be needed.

## Supplementary Information

**Additional file 1.** PRISMA checklists.

**Additional file 2.** Search strategies.

**Additional file 3.** List of excluded studies after full-text screening.

**Additional file 4.** Forest plots, table. Risk of bias of included randomized controlled trials (a, b) and non-randomized studies (c, d). (a) Risk of bias graph of randomized controlled trials: review authors' judgements about each risk of bias item presented as percentages across all included studies. (b) Risk of bias graph of randomized controlled trials: review authors' judgements about each risk of bias item for each included study. (c) Risk of bias graph of non-randomized studies: review authors' judgements about each risk of bias item presented as percentages across all included studies. (d) Risk of bias graph of non-randomized studies: review authors' judgements about each risk of bias item for each included study. **Figure S2**. Contour-enhanced funnel plot for mortality of non-intubation patients in non-randomized studies. **Figure S3**. Contour-enhanced funnel plot for intubation rate of non-intubation patients in non-randomized studies. **Figure S4**. Adverse events in randomized controlled trials. **Table S1**. Adverse events in non-randomized studies. **Figure S5**. Hospital length of stay of non-intubated patients. **Figure S6**. ICU length of stay of non-intubated patients. **Figure S7**. ICU-free days of non-intubated patients in randomized controlled trials. **Figure S8**. Ventilator-free days of non-intubated patients. **Figure S9**. Ventilator-free days of intubated patients in non-randomized studies.

## Data Availability

The datasets used and/or analyzed during the current study are available from the corresponding author upon reasonable request.

## Abbreviations

ARDS: Acute respiratory distress syndrome
CI: Confidence interval
COT: Conventional oxygen therapy
GRADE: Grading of Recommendations, Assessment, Development and Evaluation
HFNC: High-flow nasal cannula
ICU: Intensive care unit
IMV: Invasive mechanical ventilation
LOS: Length of stay
MD: Mean differences
NIV: Non-invasive ventilation
NRS: Nonrandomized study
PRISMA: Preferred Reporting Items for Systematic Reviews and Meta-Analyses
RCT: Randomized clinical trial
RoB: Cochrane risk-of-bias tool
RoBANS: Risk of Bias Assessment tool for Nonrandomized studies
RR: Relative risk
