# Supplementary material for: Efficacy and safety of prone position in COVID-19 patients with respiratory failure: a systematic review and meta-analysis
Source: Eur J Med Res. 2022 Dec 27;27:310. doi: 10.1186/s40001-022-00953-z (PMC9792321; doi:10.1186/s40001-022-00953-z)
Supplement: Supplementary file 3 — Additional file 3. List of excluded studies after full-text screening. [file 40001_2022_953_MOESM3_ESM.docx]

**Additional file 2. List of excluded studies after full-text screening**

| **No** | **Excluded studies** | **Reason for exclusion** |
| --- | --- | --- |
| 1 | Actrn. A Study of Lying Face Down to Help Improve Oxygen Levels of Adults Not Needing Ventilators in Intensive Care with COVID-19. http://wwwwhoint/trialsearch/Trial2aspx?TrialID=ACTRN12620000740998. 2020 | Ongoing study |
| 2 | Aliano Pina M, Ruiz C, Monedero P, Galan J. Cardiopulmonary resuscitation during the COVID-19 pandemic in Spain. [Spanish]. Revista espanola de anestesiologia y reanimacion. 2020;16 | Duplicate |
| 3 | Aliaño Piña M, Ruiz C, Monedero P, Galán J. [Cardiopulmonary resuscitation during the COVID-19 pandemic in Spain]. Rev Esp Anestesiol Reanim (Engl Ed). 2020 | Other than English or Korean |
| 4 | Allicock KA, Coyne D, Garton AN, Hare EC, Seckel MA. Crit Care Nurse. 2021:e1-e11 | Study other than randomized controlled trials or observational studies |
| 5 | Alsharif H, Belkhouja K. Feasibility and efficacy of prone position combined with cpap in COVID-19 patients with AHRF. Critical Care Medicine. 2021;49 (1 SUPPL 1):120 | Study other than randomized controlled trials or observational studies |
| 6 | Althunayyan S, Almutary AM, Junaidallah MA, Heji AS, Almazroua F, Alsofayan YM, et al. Prone position protocol in awake COVID-19 patients: A prospective study in the emergency department. Journal of Infection and Public Health. 2022;22:22 | Study not compared to non-prone position |
| 7 | Altinay M, Sayan I, Turk HS, Cinar AS, Sayin P, Yucel T, et al. Effect of early awake prone positioning application on prognosis in patients with acute respiratory failure due to COVID-19 pneumonia: a retrospective observational study. Braz J Anesthesiol. 2021;16:16 | Study other than randomized controlled trials or observational studies |
| 8 | Ates I, Erden A, Gurler EK, Caglayan A, Gucbey O, Karakas O, et al. Compliance to not only prone but also lateral and supine positioning improves outcome in hospitalised COVID-19 patients. International Journal of Clinical Practice. 2021;75(11):e14673 | Study without prone position in the intervention group |
| 9 | Badrising SK, Keijmel SP, Kok B. [Awake prone positioning in covid-19 patients]. Ned Tijdschr Geneeskd. 2021;165 | Other than English or Korean |
| 10 | Bahloul M, Kharrat S, Chtara K, Hafdhi M, Turki O, Baccouche N, et al. Clinical characteristics and outcomes of critically ill COVID-19 patients in Sfax, Tunisia. Acute & Critical Care. 2022;37(1):84-93 | Study not compared to non-prone position |
| 11 | Bahloul M, Kharrat S, Hfidhi M, Maalla A, Turki O, Chtara K, et al. Impact of prone position on outcomes of COVID-19 patients with spontaneous breathing. Acute Crit. 2021;12:12 | Duplicate |
| 12 | Bansbach J, Wenz J, Kaufmann K, Heinrich S, Kalbhenn J. Sevoflurane in combination with esketamine is an effective sedation regimen in COVID-19 patients enabling assisted spontaneous breathing even during prone positioning. Anestezjologia Intensywna Terapia. 2022;54(1):23-9 | Study not compared to non-prone position |
| 13 | Bell J, William Pike C, Kreisel C, Sonti R, Cobb N. Predicting Impact of Prone Position on Oxygenation in Mechanically Ventilated Patients with COVID-19. Journal of Intensive Care Medicine. 2022:8850666221081757 | Study not compared to non-prone position |
| 14 | Bisso IC, Huespe I, Lockhart C, Masso A, Anaya JG, Hornos M, et al. COVID-19 in the intensive care unit. Analysis of the experience during the first month of pandemic. [Spanish]. Medicina (Argentina). 2020;80(Supplement 3):25-30 | Other than English or Korean |
| 15 | Carpio-Orantes LD, Gonzalez-Segovia O, Mojica-Rios F, Suarez-Mandujano MP, Martinez-Rojas M, Cortes-Roman JS, et al. Severe pneumonia due to COVID-19 cured with conscious proning and tocilizumab. A report of a case and review of the pharmacological therapeutic evidence. [Spanish]. Medicina Interna de Mexico. 2021;34(4):585-95 | Other than English or Korean |
| 16 | Carrillo Hernandez-Rubio J, Sanchez-Carpintero Abad M, Yordi Leon A, Doblare Higuera G, Garcia Rodriguez L, Garcia Torrejon C, et al. Outcomes of an intermediate respiratory care unit in the COVID-19 pandemic. PLoS One. 2020;15(12):e0243968 | Study not compared to non-prone position |
| 17 | Ctri. Comparison of two different types of simple non-invasive oxygen therapy devices in coronavirus lung infection. http://wwwwhoint/trialsearch/Trial2aspx?TrialID=CTRI/2020/07/026835. 2020 | Ongoing study |
| 18 | Ctri. To compare the effect of lying in face down position along with oxygen therapy given through high flow nasal cannula device versus lying face down with oxygen given by non-rebreathing face mask in COVID-19 patients. http://wwwwhoint/trialsearch/Trial2aspx?TrialID=CTRI/2020/12/029587. 2020 | Ongoing study |
| 19 | Ctri. Self-prone positioning to reduce need of ventilatory support in COVID-19 patients. http://wwwwhoint/trialsearch/Trial2aspx?TrialID=CTRI/2020/12/029898. 2020 | Ongoing study |
| 20 | Cunha MCA, Schardong J, Righi NC, Lunardi AC, Sant'Anna GN, Isensee LP, et al. Impact of prone positioning on patients with COVID-19 and ARDS on invasive mechanical ventilation: a multicenter cohort study. Jornal Brasileiro De Pneumologia: Publicacao Oficial Da Sociedade Brasileira De Pneumologia E Tisilogia. 2022;48(2):e20210374 | Study not compared to non-prone position |
| 21 | Dalpiaz G, Gamberini L, Carnevale A, Spadaro S, Mazzoli CA, Piciucchi S, et al. Clinical implications of microvascular CT scan signs in COVID-19 patients requiring invasive mechanical ventilation. Radiologia Medica. 2022;127(2):162-73 | Study not compared to non-prone position |
| 22 | Damiani E, Casarotta E, Carsetti A, Mariotti G, Vannicola S, Giorgetti R, et al. Too much tolerance for hyperoxemia in mechanically ventilated patients with SARS-CoV-2 pneumonia? Report from an Italian intensive care unit. Frontiers in Medicine. 2022;9:957773 | Study not reported about efficacy and safety of treatment |
| 23 | Di Mascio N, Clarke S, de Loughry G, Altaf W. Prone positioning for mechanically ventilated patients with coronavirus disease 2019: the experience of an Irish regional hospital intensive care unit. Irish Journal of Medical Science. 2022;11:11 | Study not compared to non-prone position |
| 24 | Ehrmann S, Li J, Ibarra-Estrada M, Perez Y, Pavlov I, McNicholas B, et al. Awake prone positioning for COVID-19 acute hypoxaemic respiratory failure: a randomised, controlled, multinational, open-label meta-trial. Lancet Respir Med. 2021;20:20 | Study other than randomized controlled trials or observational studies |
| 25 | Engström K, Saber A. [Patient treated for severe Covid-19 with paralysis of cranial nerves]. Lakartidningen. 2021;118 | Other than English or Korean |
| 26 | Euctr FR. Low dose of IL-2 In Acute respiratory DistrEss syndrome related to COVID-19. http://wwwwhoint/trialsearch/Trial2aspx?TrialID=EUCTR2020-001571-32-FR. 2020 | Ongoing study |
| 27 | Fazzini B, Fowler AJ, Zolfaghari P. Effectiveness of prone position in spontaneously breathing patients with COVID-19: A prospective cohort study. Journal of the Intensive Care Society. 2021 | Study not compared to non-prone position |
| 28 | Fazzini B, Fowler AJ, Zolfaghari P. Effectiveness of prone position in spontaneously breathing patients with COVID-19: A prospective cohort study. The Journal of the Intensive Care Society. 2022;23(3):362-5 | Study not compared to non-prone position |
| 29 | Ferrando C, Mellado-Artigas R, Gea A, Arruti E, Aldecoa C, Adalia R, et al. Awake prone positioning does not reduce the risk of intubation in COVID-19 treated with high-flow nasal oxygen therapy: a multicenter, adjusted cohort study. Crit Care. 2020;24(1):597 | Duplicate |
| 30 | Garcia MA, Rampon GL, Doros G, Jia S, Jagan N, Gillmeyer K, et al. Rationale and Design of the Awake Prone Position for Early Hypoxemia in COVID-19 Study Protocol: A Clinical Trial. Ann Am Thorac Soc. 2021;18(9):1560-6 | Ongoing study |
| 31 | Garcia-Rodriguez DF, Martinez-Resendiz JA, Gonzalez-Juarez F. A priori accept that mechanical ventilation in the prone position in COVID-19 is the best?. [Spanish]. Neumologia y Cirugia de Torax(Mexico). 2020;79(3):141-4 | Other than English or Korean |
| 32 | Haller ML, Brunner ME, Zender HO. Intensive care unit treatment for patients suffering from COVID-19: The neuchatel experience. [French]. Rev Med Suisse. 2020;16(716):2284-6 | Duplicate |
| 33 | Haller ML, Brunner ME, Zender HO. [Intensive care unit treatment for patients suffering from COVID-19: the Neuchâtel experience]. Rev Med Suisse. 2020;16(716):2284-6 | Other than English or Korean |
| 34 | Hallifax RJ, Porter BM, Elder PJ, Evans SB, Turnbull CD, Hynes G, et al. Successful awake proning is associated with improved clinical outcomes in patients with COVID-19: single-centre high-dependency unit experience. BMJ Open Respir Res. 2020;7(1) | Study not compared to non-prone position |
| 35 | Hanisch V. COVID-19: The role of intensive care. [German]. Public Health Forum. 2021;29(1):11-4 | Other than English or Korean |
| 36 | Herbstreit F, Welsner M, Taube C, Ernst EC, Stoppler T, Brenner T, et al. ECMO as a bridging to recovery in a 19-year-old patient with e-cigarette / vaping-product associated lung injury (EVALI). [German]. Anasthesiologie und Intensivmedizin. 2020;61(12):605-8 | Other than English or Korean |
| 37 | Hernandez-Rubio JC, Abad MSC, Leon AY, Higuera GD, Rodriguez LG, Torrejon CG, et al. Outcomes of an intermediate respiratory care unit in the COVID-19 pandemic. PLoS One. 2020;15 (12 December) (no pagination)(e0243968) | Study not compared to non-prone position |
| 38 | Hu Y, Li X, Gong W. Nutritional support for critically ill patients with COVID-19. [Chinese]. Zhejiang da xue xue bao. 2020;Yi xue ban = Journal of Zhejiang University. Medical sciences. 49(3):347-55 | Duplicate |
| 39 | Hu Y, Li X, Gong W. [Nutritional support for critically ill patients with COVID-19]. Zhejiang Da Xue Xue Bao Yi Xue Ban. 2020;49(3):347-55 | Other than English or Korean |
| 40 | Irct20160126026217N. Investigation of the effects of prone position on cardiac and respiratory status in patients with Coronavirus. http://wwwwhoint/trialsearch/Trial2aspx?TrialID=IRCT20160126026217N4. 2020 | Ongoing study |
| 41 | Isrctn. Therapeutic plasma exchange (removal of the non-cell portion of blood) in critically ill adult patients with serious SARS CoV-2 disease (COVID-19). http://wwwwhoint/trialsearch/Trial2aspx?TrialID=ISRCTN21363594. 2020 | Ongoing study |
| 42 | Isrctn. Could prone positioning increase lung function for patients with severe COVID-19 infection? http://wwwwhoint/trialsearch/Trial2aspx?TrialID=ISRCTN54917435. 2020 | Ongoing study |
| 43 | Jakob SM, Michel K, Kindler A. [COVID-19 in the Intensive Care Unit: Medical, Nursing, and Physical Therapy Challenges]. Praxis (Bern 1994). 2021;110(9):512-6 | Other than English or Korean |
| 44 | Janos T. ECG pearl: Prone position ECG for COVID-19 patients. [Hungarian]. Orv Hetil. 2020;161(26):1103-4 | Other than English or Korean |
| 45 | k66ft RBR. Randomized, study to evaluate early prone position in patients with COVID-19. http://wwwwhoint/trialsearch/Trial2aspx?TrialID=RBR-2k66ft. 2020 | Ongoing study |
| 46 | Kremeier P, Pulletz S, Woll C, Oczenski W, Bohm S. Arbeitsprozesse bei beatmungspatienten mit kritischen keimspektren. [German]. Intensiv- und Notfallbehandlung. 2020;45(2):42-71 | Other than English or Korean |
| 47 | Lahera T, Ruiz C, Aquevedo A, Cotoras P, Uribe J, Montenegro C. [Features of 50 patients with SARS-CoV2 admitted to an intensive care unit and requiring mechanical ventilation]. Rev Med Chil. 2020;148(12):1725-33 | Other than English or Korean |
| 48 | Langer T, Brioni M, Guzzardella A, Carlesso E, Cabrini L, Castelli G, et al. Prone position in intubated, mechanically ventilated patients with COVID-19: a multi-centric study of more than 1000 patients. Crit Care. 2021;25(1):128 | Study not compared to non-prone position |
| 49 | Le Terrier C, Sigaud F, Lebbah S, Desmedt L, Hajage D, Guerin C, et al. Early prone positioning in acute respiratory distress syndrome related to COVID-19: a propensity score analysis from the multicentric cohort COVID-ICU network-the ProneCOVID study. Critical Care (London, England). 2022;26(1):71 | Study not compared to non-prone position |
| 50 | Liu X, Liu H, Lan Q, Zheng X, Duan J, Zeng F. Early prone positioning therapy for patients with mild COVID-19 disease. Med Clin (Barc). 2021;156(8):386-9 | Study involving patients without COVID-19 |
| 51 | Loureiro-Amigo J, Suarez-Carantona C, Oriol I, Sanchez-Diaz C, Coloma-Conde A, Manzano-Espinosa L, et al. Prone Position in COVID-19 Patients With Severe Acute Respiratory Distress Syndrome Receiving Conventional Oxygen Therapy: A Retrospective Study. Archivos de Bronconeumologia. 2022;58(3):277-80 | Duplicate |
| 52 | Mathews KS, Soh H, Shaefi S, Wang W, Bose S, Coca S, et al. Prone Positioning and Survival in Mechanically Ventilated Patients with Coronavirus Disease 2019-Related Respiratory Failure*. Crit Care Med. 2021:1026-37 | Study not compared to non-prone position |
| 53 | Melamed R, Paz F, Jepsen S, Smith C, Saavedra R, Mulder M, et al. Prognostic factors and outcomes in COVID-19 patients requiring prolonged mechanical ventilation: a retrospective cohort study. Therapeutic Advances in Respiratory Disease. 2022;16:17534666221086415 | Study not reported about efficacy and safety of treatment |
| 54 | Meredith S, Bhat P, Ahmed MA, Singh K. A retrospective analysis of the effect of self proning on disease progression in COVID-19 patients. American Journal of Respiratory and Critical Care Medicine Conference: American Thoracic Society International Conference, ATS. 2021;203(9) | Study other than randomized controlled trials or observational studies |
| 55 | Nauka PC, Chekuri S, Aboodi M, Hope AA, Gong MN, Chen JT. A Case-Control Study of Prone Positioning in Awake and Nonintubated Hospitalized Coronavirus Disease 2019 Patients. Crit Care Explor. 2021;3(2):e0348 | Study involving patients without COVID-19 |
| 56 | Nct. SMP vs RIRS for Symptomatic Lower Pole Renal Calculi of 10-20 mm Size: a Randomized Controlled Trial. https://clinicaltrialsgov/show/NCT02519634. 2015 | Ongoing study |
| 57 | Nct. Steroids and Unfractionated Heparin in Critically Ill Patients With Pneumonia From COVID-19 Infection. https://clinicaltrialsgov/show/NCT04528888. 2020 | Ongoing study |
| 58 | Nct. Convalescent Plasma as a Possible Treatment for COVID-19. https://clinicaltrialsgov/show/NCT04442191. 2020 | Ongoing study |
| 59 | Nct. Awake Pronation for Covid-19 Treatment. https://clinicaltrialsgov/show/NCT04667286. 2020 | Ongoing study |
| 60 | Nct. COVID-19 Watch + COVID-19 Pulse. https://clinicaltrialsgov/show/NCT04581863. 2020 | Ongoing study |
| 61 | Nct. Fibrinolytic Therapy to Treat ARDS in the Setting of COVID-19 Infection: a Phase 2a Clinical Trial. https://clinicaltrialsgov/show/NCT04357730. 2020 | Ongoing study |
| 62 | Nct. Sarilumab for Patients With Moderate COVID-19 Disease. https://clinicaltrialsgov/show/NCT04359901. 2020 | Ongoing study |
| 63 | Nct. Hydroxychloroquine in SARS-CoV-2 (COVID-19) Pneumonia Trial. https://clinicaltrialsgov/show/NCT04382625. 2020 | Ongoing study |
| 64 | Nct. Early Versus Late ECMO Therapy in COVID-19 Induced ARDS (ECMO-VID). https://clinicaltrialsgov/show/NCT04341285. 2020 | Ongoing study |
| 65 | Nct. Angiotensin-(1,7) Treatment in COVID-19: the ATCO Trial. https://clinicaltrialsgov/show/NCT04332666. 2020 | Ongoing study |
| 66 | Nct. COVID-19 Patient Positioning Pragmatic Trial. https://clinicaltrialsgov/show/NCT04359797. 2020 | Ongoing study |
| 67 | Nct. GM-CSF Inhalation to Prevent ARDS in COVID-19 Pneumonia. https://clinicaltrialsgov/show/NCT04569877. 2020 | Ongoing study |
| 68 | Nct. Accelerated Prone Position Ventilation of Patients With COVID-19. https://clinicaltrialsgov/show/NCT04384900. 2020 | Ongoing study |
| 69 | Nct. Early PP With HFNC Versus HFNC in COVID-19 Induced Moderate to Severe ARDS. https://clinicaltrialsgov/show/NCT04325906. 2020 | Ongoing study |
| 70 | Nct. HFNT vs. COT in COVID-19. https://clinicaltrialsgov/show/NCT04655638. 2020 | Ongoing study |
| 71 | Nct. Awake Prone Position to Reduce Ventilation Inhomogeneity in COVID-19 Acute Respiratory Failure. https://clinicaltrialsgov/show/NCT04632602. 2020 | Ongoing study |
| 72 | Nct. Trial of Therapeutic Hypothermia in Patients With ARDS. https://clinicaltrialsgov/show/NCT04545424. 2020 | Ongoing study |
| 73 | Nct. Awake Prone Position in Hypoxemic Patients With Coronavirus Disease 19 (COVI-PRONE): a Randomized Clinical Trial. https://clinicaltrialsgov/show/NCT04350723. 2020 | Ongoing study |
| 74 | Nct. Awake Prone Positioning to Reduce Invasive VEntilation in COVID-19 Induced Acute Respiratory failurE. https://clinicaltrialsgov/show/NCT04347941. 2020 | Ongoing study |
| 75 | Nct. Prone Positioning in Awake Patients With COVID-19 Requiring Hospitalization. https://clinicaltrialsgov/show/NCT04368000. 2020 | Ongoing study |
| 76 | Nct. Prone Position and Respiratory Outcomes in Non-Intubated COVID PatiEnts The "PRONE" Study. https://clinicaltrialsgov/show/NCT04517123. 2020 | Ongoing study |
| 77 | Nct. Effect of Prone Positioning Combined With High Flow Oxygen Therapy on Oxygenation During Acute Respiratory Failure Due to COVID-19. https://clinicaltrialsgov/show/NCT04543760. 2020 | Ongoing study |
| 78 | Nct. PROne Positioning in coVID-19 Oxygeno-dependent Patients in Spontaneous Ventilation (PROVID Study). https://clinicaltrialsgov/show/NCT04366856. 2020 | Ongoing study |
| 79 | Nct. Prone Positioning in Non-intubated Patients With COVID-19 Associated Acute Respiratory Failure. https://clinicaltrialsgov/show/NCT04477655. 2020 | Ongoing study |
| 80 | Nct. COVID-19 smArtphone-based Trial of Non-ICU Admission Prone Positioning. https://clinicaltrialsgov/show/NCT04344587. 2020 | Ongoing study |
| 81 | Nct. Prone Position in Patients on High-flow Nasal Oxygen Therapy for COVID-19 (HIGH-PRONE-COVID-19). https://clinicaltrialsgov/show/NCT04358939. 2020 | Ongoing study |
| 82 | Nct. Prolonged Prone Positioning for COVID-induced Acute Respiratory Distress Syndrome (ARDS): a Pilot Study. https://clinicaltrialsgov/show/NCT04581811. 2020 | Ongoing study |
| 83 | Nct. CORONA (COvid pRONe hypoxemiA): prone Positioning for Hypoxemic COVID-19 Patients With Do-not-intubate Goals. https://clinicaltrialsgov/show/NCT04402879. 2020 | Ongoing study |
| 84 | Nct. COVid-19: awake Proning and High-flow Nasal Cannula in respiratorY DistrEss. https://clinicaltrialsgov/show/NCT04395144. 2020 | Ongoing study |
| 85 | Nct. Awake-Prone Positioning Strategy for Hypoxic Patients With COVID-19. https://clinicaltrialsgov/show/NCT04547283. 2020 | Ongoing study |
| 86 | Nct. Prone Positioning and High-flow Nasal Cannula in COVID-19 Induced ARDS. https://clinicaltrialsgov/show/NCT04391140. 2020 | Ongoing study |
| 87 | Nct. Breathing Effort in Covid-19 Pneumonia: effects of Positive Pressure, Inspired Oxygen Fraction and Decubitus. https://clinicaltrialsgov/show/NCT04885517. 2021 | Ongoing study |
| 88 | Nct. Self-prone Positioning for Awake Non-intubated Patients With COVID-19. https://clinicaltrialsgov/show/NCT04760561. 2021 | Ongoing study |
| 89 | Nct. Awake Prone Positioning in COVID-19 Suspects With Hypoxemic Respiratory Failure. https://clinicaltrialsgov/show/NCT04853979. 2021 | Ongoing study |
| 90 | Nct. Intravenous Imatinib in Mechanically Ventilated COVID-19 Patients. https://clinicaltrialsgov/show/NCT04794088. 2021 | Ongoing study |
| 91 | Nl. Intensive Care Unit specific Virtual Reality (ICU-VR) to improve psychological impairments in survivors of COVID-19; a multicentre, randomised controlled trial. http://wwwwhoint/trialsearch/Trial2aspx?TrialID=NL8835. 2020 | Ongoing study |
| 92 | Pan C, Zhang W, Du B, Qiu HB, Huang YZ. [Prone ventilation for coronavirus disease 2019: an urgent salvage therapy]. Zhonghua Nei Ke Za Zhi. 2020;59(9):670-2 | Other than English or Korean |
| 93 | Perez-Nieto OR, Escarraman-Martinez D, Guerrero-Gutierrez MA, Zamarron-Lopez EI, Mancilla-Galindo J, Kammar-Garcia A, et al. Awake prone positioning and oxygen therapy in patients with COVID-19: the APRONOX study. European Respiratory Journal. 2022;59(2):02 | Duplicate |
| 94 | Qiu HB, Li XY, Du B, Kang HYJ, Wang YS, Wang F, et al. [The keypoints in treatment of the critical coronavirus disease 2019 patient(1)]. Zhonghua Jie He He Hu Xi Za Zhi. 2020;43(4):273-7 | Other than English or Korean |
| 95 | Rampon G, Jia S, Agrawal R, Arnold N, Martin-Quiros A, Fischer EA, et al. Smartphone-Guided Self-prone Positioning vs Usual Care in Nonintubated Hospital Ward Patients With COVID-19: A Pragmatic Randomized Clinical Trial. Chest. 2022;18:18 | Study involving patients without COVID-19 |
| 96 | Rosen J, von Oelreich E, Fors D, Jonsson Fagerlund M, Taxbro K, Skorup P, et al. Awake prone positioning in patients with hypoxemic respiratory failure due to COVID-19: the PROFLO multicenter randomized clinical trial. Critical Care. 2021;25 (1) (no pagination)(209) | Duplicate |
| 97 | Schellhaaß A, Pöselt S, Schwietring J, Horter J, Münzberg M. [Air ambulance intensive care transport in prone position for COVID-19 ARDS]. Notf Rett Med. 2020:1-5 | Other than English or Korean |
| 98 | Silva Junior JM, Treml RE, Golinelli PC, Segundo M, Menezes PFL, Umada JDA, et al. Response of patients with acute respiratory failure caused by COVID-19 to awake-prone position outside the intensive care unit based on pulmonary involvement. Clinics (Sao Paulo, Brazil). 2021;76:e3368 | Study not compared to non-prone position |
| 99 | Simioli F, Annunziata A, Langella G, Martino M, Musella S, Fiorentino G. Early Prone Positioning and Non-Invasive Ventilation in a Critical COVID-19 Subset. A Single Centre Experience in Southern Italy. Turk Thorac J. 2021;22(1):57-61 | Study not compared to non-prone position |
| 100 | Sosa-Garcia JO, Gutierrez-Villasenor AO, Garcia-Briones A, Romero-Gonzalez JP, Juarez-Hernandez E, Gonzalez-Chon O. Experience in the management of severe COVID-19 patients in an intensive care unit. [Spanish]. Cirugia y Cirujanos (English Edition). 2020;88(5):569-75 | Other than English or Korean |
| 101 | Taboada M, González M, Álvarez A, González I, García J, Eiras M, et al. Effectiveness of Prone Positioning in Nonintubated Intensive Care Unit Patients With Moderate to Severe Acute Respiratory Distress Syndrome by Coronavirus Disease 2019. Anesth Analg. 2021;132(1):25-30 | Study not compared to non-prone position |
| 102 | Thompson AE, Ranard BL, Wei Y, Jelic S. Prone Positioning in Awake, Nonintubated Patients With COVID-19 Hypoxemic Respiratory Failure. JAMA Intern Med. 2020;180(11):1537-9 | Study other than randomized controlled trials or observational studies |
| 103 | Tomcsányi J. [ECG pearl: prone position ECG for COVID-19 patients]. Orv Hetil. 2020;161(26):1103-4 | Other than English or Korean |
| 104 | Tonelli R, Pisani L, Tabbi L, Comellini V, Prediletto I, Fantini R, et al. Early awake proning in critical and severe COVID-19 patients undergoing noninvasive respiratory support: A retrospective multicenter cohort study. Pulmonology. 2022;28(3):181-92 | Duplicate |
| 105 | Wei Z, Chun P, Qing S. We should pay close attention to some issues in the process of lung injury therapy of critical severe COVID-19. [Chinese]. Medical Journal of Chinese People's Liberation Army. 2020;45(3):236-40 | Other than English or Korean |
| 106 | Wong MJ, Bharadwaj S, Lankford AS, Galey JL, Kodali BS. Mechanical ventilation and prone positioning in pregnant patients with severe COVID-19 pneumonia: experience at a quaternary referral center. International Journal of Obstetric Anesthesia. 2022;49:103236 | Study not compared to non-prone position |
| 107 | Wrigge H, Glien C. Specific treatment of acute lung failure. [German]. Anaesthesist. 2020;69(11):847-56 | Duplicate |
| 108 | Wrigge H, Glien C. [Specific treatment of acute lung failure]. Anaesthesist. 2020;69(11):847-56 | Other than English or Korean |
| 109 | Xu Y, Meng M, Liu J, Chen D. [Practical procedure of prone position ventilation in critical coronavirus disease 2019 patients]. Zhonghua Wei Zhong Bing Ji Jiu Yi Xue. 2021;33(4):393-8 | Duplicate |
| 110 | Yan X, Mei M, Jiao L, Dechang C. Practical procedure of prone position ventilation in critical coronavirus disease 2019 patients. [Chinese]. Zhonghua Wei Zhong Bing Ji Jiu Yi Xue. 2021;33(4):393-8 | Other than English or Korean |
| 111 | Zaaqoq AM, Barnett AG, Griffee MJ, MacLaren G, Jacobs JP, Heinsar S, et al. Beneficial Effect of Prone Positioning During Venovenous Extracorporeal Membrane Oxygenation for Coronavirus Disease 2019. Critical Care Medicine. 2021;27:27 | Study without prone position in the intervention group |
| 112 | Zaaqoq AM, Barnett AG, Griffee MJ, MacLaren G, Jacobs JP, Heinsar S, et al. Beneficial Effect of Prone Positioning During Venovenous Extracorporeal Membrane Oxygenation for Coronavirus Disease 2019. Critical Care Medicine. 2021;27:27 | Duplicate |
