# Supplementary material for: Efficacy and safety of prone position in COVID-19 patients with respiratory failure: a systematic review and meta-analysis
Source: Eur J Med Res. 2022 Dec 27;27:310. doi: 10.1186/s40001-022-00953-z (PMC9792321; doi:10.1186/s40001-022-00953-z)

**Figure S1. Risk of bias of included randomized controlled trials (a, b) and non-randomized studies (c, d)**

**(a) Risk of bias graph of randomized controlled trials: review authors' judgements about each risk of bias item presented as percentages across all included studies**


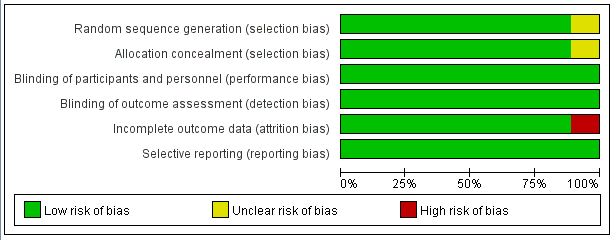


**(b) risk of bias graph of randomized controlled trials: review authors' judgements about each risk of bias item for each included study**


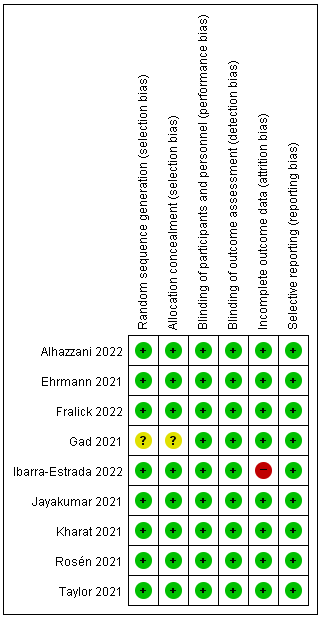


**(c) Risk of bias graph of non-randomized studies: review authors' judgements about each risk of bias item presented as percentages across all included studies**


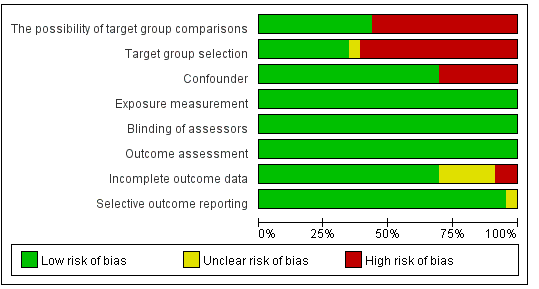


**(d) Risk of bias graph of non-randomized studies: review authors' judgements about each risk of bias item for each included study**


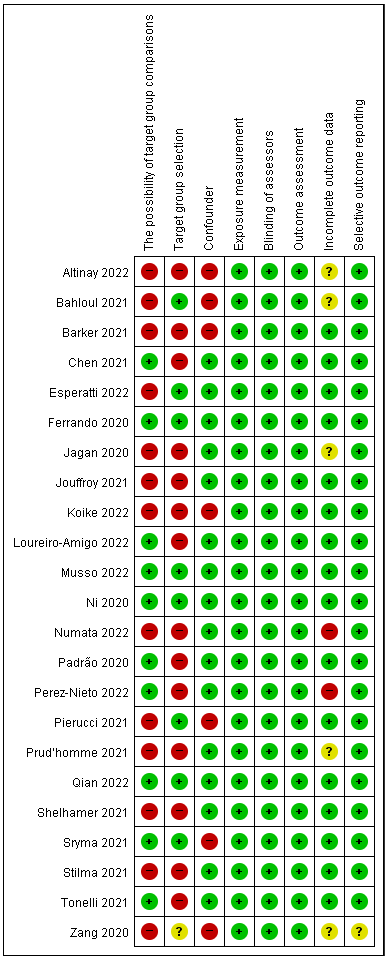


**Figure S2. Contour-enhanced funnel plot for mortality of non-intubation patients in non-randomized studies**

**
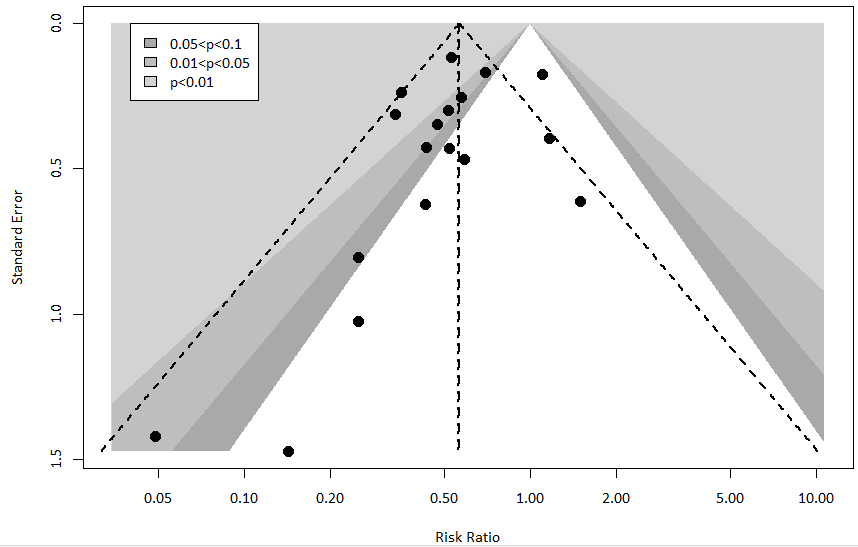
**

**Figure S3. Contour-enhanced funnel plot for intubation rate of non-intubation patients in non-randomized studies**

**
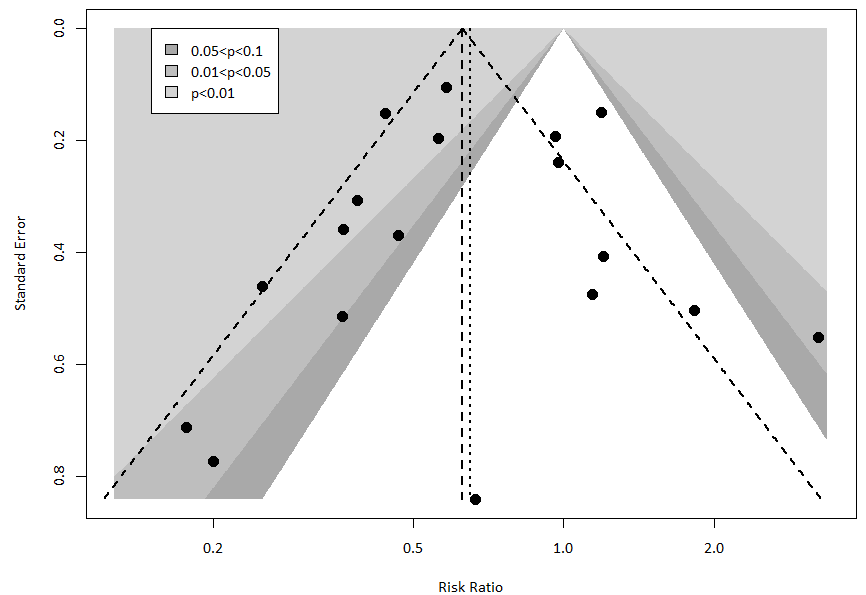
**

**Figure S4. Adverse events in randomized controlled trials**

**
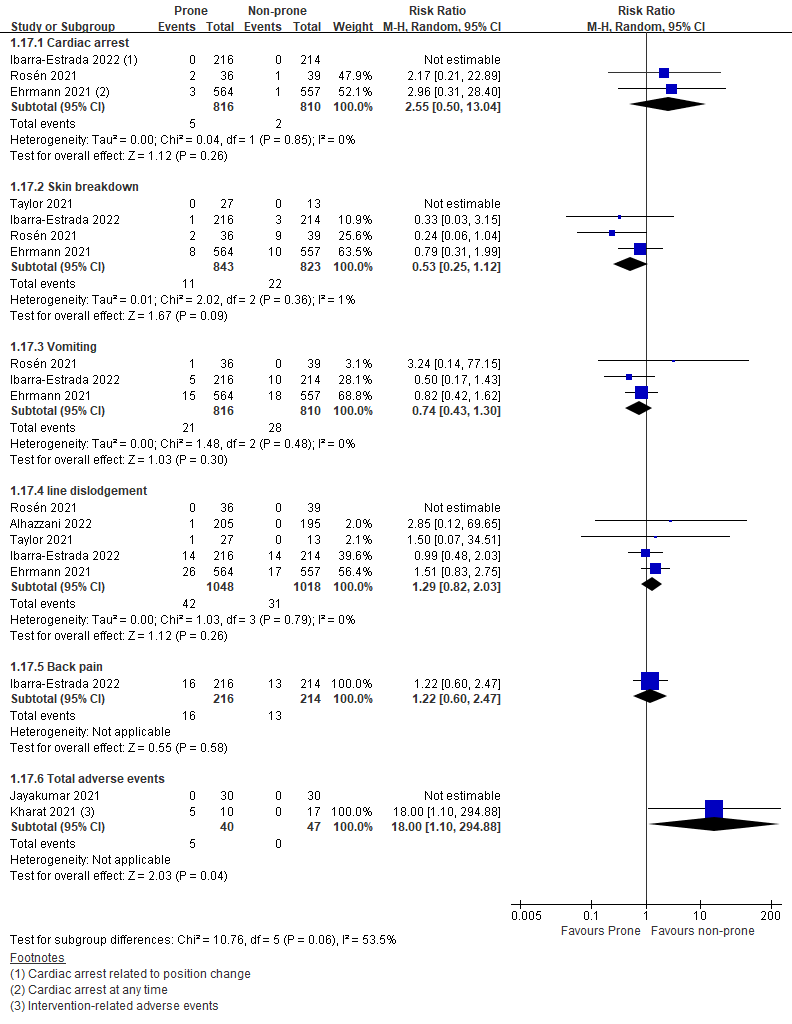
**

**Table S1. Adverse events in non-randomized studies**

| Adverse events | Cases in the prone group | References |
| --- | --- | --- |
| **Non-intubated patients** | | |
| Desaturation or hemodynamic worsening | 0/30 | Sryma et al. (2021) [34] |
| Back pain | 2/30 | Sryma et al. (2021) [34] |
|  | 3/57 | Padrão et al. (2020) [42] |
| Bloating sensation | 2/30 | Sryma et al. (2021) [34] |
| Gastric distension and vomit | 0/81 | Musso et al. (2022) [30] |
| Peripheral line removal | 2/57 | Padrão et al. (2020) [42] |
|  | 2/81 | Musso et al. (2022) [30] |
| Nasal skin ulceration | 2/81 | Musso et al. (2022) [30] |
| Major adverse events | 0/48 | Prud’homme et al. (2021) [44] |
| Overall adverse events | 0/17 | Ni et al. (2020) [5] |
|  | 0/38 | Tonelli et al. (2021) [46] |
| **Intubated patients** | | |
| Endotracheal tube dislocation | 0/62 | Shelhamer et al. (2021) [9] |
| Peripheral line removal | 2/62 | Shelhamer et al. (2021) [9] |

**Figure S5. Hospital length of stay of non-intubated patients**

**
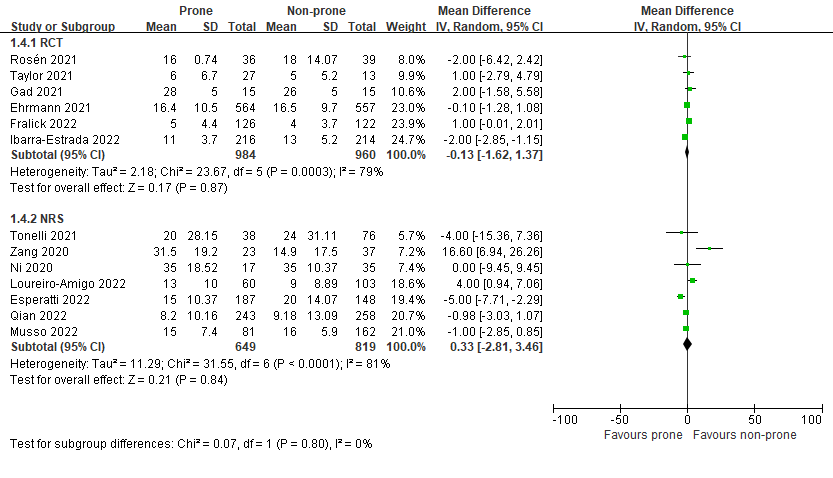
**

**Figure S6. ICU length of stay of non-intubated patients**

**
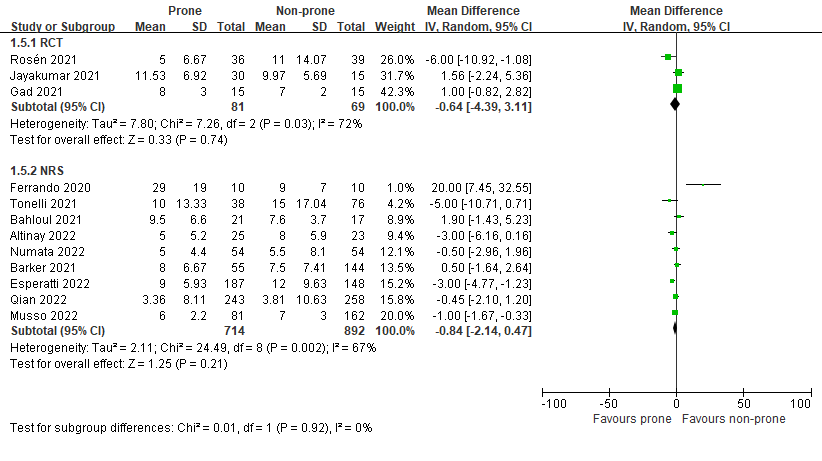
**

**Figure S7. ICU-free days of non-intubated patients in randomized controlled trials**

**
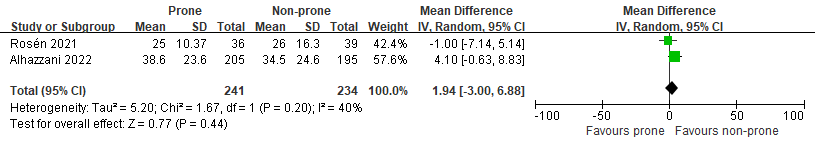
**

**Figure S8. Ventilator-free days of non-intubated patients**

**
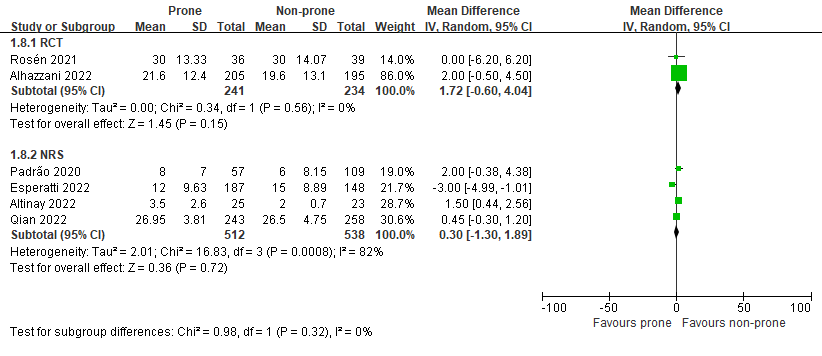
**

**Figure S9. Ventilator-free days of intubated patients in non-randomized studies**


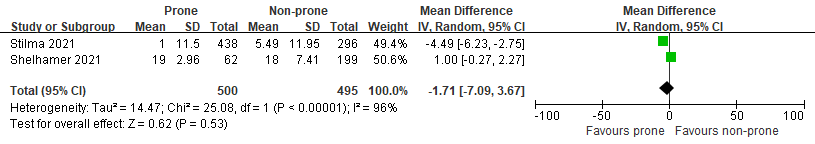

Supplement: Supplementary file 4 — Additional file 4. Forest plots, table. Risk of bias of included randomized controlled trials (a, b) and non-randomized studies (c, d). (a) Risk of bias graph of randomized controlled trials: review authors' judgements about each risk of bias item presented as percentages across all included studies. (b) Risk of bias graph of randomized controlled trials: review authors' judgements about each risk of bias item for each included study. (c) Risk of bias graph of non-randomized studies: review authors' judgements about each risk of bias item presented as percentages across all included studies. (d) Risk of bias graph of non-randomized studies: review authors' judgements about each risk of bias item for each included study. Figure S2. Contour-enhanced funnel plot for mortality of non-intubation patients in non-randomized studies. Figure S3. Contour-enhanced funnel plot for intubation rate of non-intubation patients in non-randomized studies. Figure S4. Adverse events in randomized controlled trials. Table S1. Adverse events in non-randomized studies. Figure S5. Hospital length of stay of non-intubated patients. Figure S6. ICU length of stay of non-intubated patients. Figure S7. ICU-free days of non-intubated patients in randomized controlled trials. Figure S8. Ventilator-free days of non-intubated patients. Figure S9. Ventilator-free days of intubated patients in non-randomized studies. [file 40001_2022_953_MOESM4_ESM.docx]
